# Development of neutrosophic cubic hesitant fuzzy exponential aggregation operators with application in environmental protection problems

**DOI:** 10.1038/s41598-022-22399-3

**Published:** 2023-03-31

**Authors:** Ateeq Ur Rehman, Muhammad Gulistan, Mumtaz Ali, Mohammed M. Al-Shamiri, Shahab Abdulla

**Affiliations:** 1 Department of Mathematics and Statistics, Hazara University Mansehra, Khyber Pakhtunkhwa, Pakistan; 2 UniSQ College, University of Southern Queensland, Darling Heights, QLD 4300 Australia; 3 Department of Mathematics, Faculty of Science and Arts, Muhayl Asser, King Khalid University, Abha, Kingdom of Saudi Arabia; 4 Department of Mathematics and Computer, Faculty of Science, Ibb University, Ibb, Yemen; 5 Department of Electrical and Computer Engineering, University of Alberta, Edmonton, Canada

**Keywords:** Mathematics and computing, Applied mathematics, Computational science

## Abstract

The population growth and urbanization has caused an exponential increase in waste material. The proper disposal of waste is a challenging problem nowadays. The proper disposal site selection with typical sets and operators may not yield fruitful results. To handle such problems, the exponential aggregation operators based on neutrosophic cubic hesitant fuzzy sets are proposed. For appropriate decisions in a decision-making problem, it is important to have a handy environment and aggregation operators. Many multi attribute decision making methods often ignore the uncertainty and hence yields the results which are not reliable. The neutrosophic cubic hesitant fuzzy set can efficiently handle the complex information in a decision-making problem, as it combines the advantages of neutrosophic cubic set and hesitant fuzzy set. In this paper first we establish exponential operational laws in neutrosophic cubic hesitant fuzzy sets, in which the exponents are neutrosophic cubic hesitant fuzzy numbers and bases are positive real numbers. In order to use neutrosophic cubic hesitant fuzzy sets in decision making, we are developing exponential aggregation operators and investigate their properties in the current study. In many multi expert decision-making methods there are different decision matrices but same weighting vector for attributes. The results of a multi expert decision-making problem becomes more reliable if every decision expert has its own decision matrix along with his own weighting vector for attributes. In this study, we are developing multi expert decision-making method that uses different weights for an attribute corresponding to different experts. At the end we present two applications of exponential aggregation operators in environmental protection multi attribute decision making problems.

## Introduction

Decision making is one of the crucial problems in real life. Aggregation operators are fundamental tools in decision making. The industrial zone site selection and solid waste disposal site selection are two important and challenging multi attribute environmental protection problems especially for developing countries. Poor management of municipal solid waste leads to environmental and water pollution which would harm to human and wildlife. Different sets and their generalizations like fuzzy set (FS), interval valued fuzzy set (IVFS), intuitionistic fuzzy set (IFS), interval intuitionistic fuzzy set (IIFS) hesitant fuzzy set (HFS), neutrosophic set (NS), neutrosophic cubic set (NCS), and several aggregation operators have been defined so for. Zadeh^1^ introduced the notion of FS as a generalization of classical set. He further extended the idea to IVFS^2^. Chen^3^ in 1992 proposed fuzzy MADM methods and discussed their applications in economics. Chen^4^ proposed the fuzzy extension of TOPSIS method for MADM problems. Xia^5^ established a novel MADM method. Chang and Wang^6^ in 2009 discussed the applications of fuzzy MADM in successful knowledge development. Attanassov^7^ introduced non-membership degree and proposed IFS. Dey et al.^8^ proposed MADM techniques in IFSs. Later the IFS was further extended to IIFS^9^. Mondal and Pramanik^10^ established intuitionistic fuzzy multi criteria group decision making approach to quality-brick selection problem. Different researchers established similarity measures and other important concepts and successfully apply their models to medical diagnosis and selection criteria. Krohling and Campanharo^11^ established different useful techniques to sort out MADM problems. Pramanik and Mondal^12^ established weighted fuzzy similarity measure based on tangent function and its application to medical diagnosis. Xu^13^ proposed some similarity measures of IFS for MADM.

Jun^14^ in 2012 combined interval value fuzzy set and fuzzy set to form cubic set. The cubic set is generalization of intuitionistic fuzzy set and interval intuitionistic fuzzy set. Cubic set become vital tool to deal the vague data. Several researchers^15–17^ explored algebraic aspects and apparently define ideal theory in cubic sets. Smarandache initiated the concept of indeterminacy and describes the notion of neutrosophic set (NS)^18^. An NS consists of three components truth, indeterminacy and falsehood. All the three components are independent of each other. This characteristic of NS enabled researchers to deal with inconsistent and vague data more efficiently. For engineering purposes, the NS is strict to [0,1] and called single valued neutrosophic set presented by Wang et al.^19^. The NS was further extended to interval neutrosophic set (INS)^20^. After the appearance of NS, researchers put their contributions in theoretical as well as technological developments of the set. Several researchers use neutrosophic and interval valued neutrosophic environments to construct MADM. Ye^21^ proposed similarity measures between INSs for MADM. Biswas et al., established useful MADM techniques using entropy and similarity measures in neutrosophic environment ^22–24^. Kharal^25^ established a multi-criteria decision making method in neutrosophic environment. Li^26^ proposed novel neutrosophic number Einstein aggregation operators for MADM problems. Mondal and Pramanik^27^ established neutrosophic decision making model for clay-brick selection in construction field based on grey relational analysis. Saha and Broumi^28^ established some new aggregation operators in INSs. Zhan et al.^29^ define aggregation operators and furnished some applications in MADM.

Torra^30^ defined hesitant fuzzy set. Hesitant fuzzy set is basically a function set on X that when applied to X returns a subset of [0,1]. Jun^31^ in 2015 introduced the concept of neutrosophic cubic set (NCS) which consists of both INS and NS. These characteristics of NCS make it a powerful tool to deal the vague and inconsistent data more efficiently. Soon after its exploration it attracted the researcher to work in many fields like medicine, algebra, engineering, decision making theory. Al-Shumrani^32^ discussed the stability analysis in neutrosophic cubic set with some applications. Cui and Ye ^33^ proposed logarithmic similarity measure of dynamic NCS and discussed their applications in medical diagnosis. Khan et al.^34^ established exponential aggregation operators in neutrosophic cubic environment and applied them in MADM problems. Later the idea of cubic hesitant fuzzy set was introduced by Mehmood et al.^35^. Ye^36,37^ established similarity measures in neutrosophic hesitant fuzzy set (NHFS) and discussed its applications in MADM. Liu and Luo^38^ established some new aggregation operators of NHFS for MADM problems. Saha et al.^39^, proposed hesitant triangular neutrosophic numbers and their applications to MADM. Liu and Shi^40^ proposed hybrid geometric aggregation operators in interval valued neutrosophic hesitant fuzzy sets and discuss its applications in MADM. Biswas et al.^41^, established useful MADM techniques using NHFSs.

Zhu et al.^42^ introduced the method of *β*-normalization to add some values to a hesitant fuzzy element (HFE), which is a useful technique in case of different cardinalities. Ye^43^ proposed new exponential operations and aggregation operators of interval neutrosophic sets for MADM. Lu and Ye^44^ introduced exponential laws in single valued neutrosophic numbers. Later the exponential aggregation operators were introduced and applied in typhoon disaster evaluation by Tan et al.^45^. Wang and Li^46^ proposed some aggregation operators in pictures hesitant fuzzy set and compared these operators with some existing decision-making methods. Tan and Zhang^47^ introduced trapezoidal fuzzy neutrosophic numbers arithmetic averaging and hybrid arithmetic averaging for MADM. Saha et al.^48^, established q-rung orthopair fuzzy weighted aggregation operators for MADM. Feng et al.^49^ define type-2 hesitant fuzzy sets and explore some important properties of these sets. Turkarslan et al.^50^, in 2021 proposed the similarity measures in fuzzy multiset with application in medical diagnosis. Saha and Makharjee^51^ defined soft interval-valued intuitionistic fuzzy rough sets and discussed some interesting properties of these sets. Senapati et al.^52^, proposed some novel interval-valued Pythagorean fuzzy aggregation operators based on Hamachar triangular norms for MADM. Recently WASPAS technique using picture fuzzy sets for MADM problems was established by Senapati et al.^53^. Wang et al.^46^, defined picture hesitant fuzzy sets and discussed their applications in MADM. Xia and Xu^5^ established novel MADM method. Several researchers^29,54–56^ established many useful techniques for MADM problems.

The NCS consider the truth, indeterminacy and falsity independently but is unable to handle the hesitant factor in each component. On the other hand, HFS is more flexible in choosing membership grades. Recently Rehman et al., defined NCHFS^57^ and geometric aggregation operators of NCHFS for MADM problems. The NCHFS can efficiently handle the complex information in a decision-making problem, as it combines the advantages of NCS and HFS. More recently, Rehman et al.^58^, established Dombi exponential aggregation operators in NCHFS and discussed their properties in solid waste disposal site selection. Also see^59,60^.

### Motivation

The industries play an important role in economic growth and prosperity of the people of a region. But there must be a need of proper planning to minimize the negative impacts of industry like pollution. Waste material is direct consequence of urbanization and population increase. The proper disposal of waste is necessary for prevention of viral diseases like typhoid, dengue and tuberculosis. The increase in population and urbanization is exponential so the exponential operational laws and aggregation operators are needed. Regarding to waste material, the information is inconsistent, incomplete and insufficient. These situations can efficiently be handled by NCHFS.

The rest of this paper is organized as follows. “Preliminaries” deals with some basic definitions used later. In “Operational laws in neutrosophic cubic hesitant fuzzy set” we discuss NCHFS and algebraic operational laws in NCHFS. In “Exponential operational laws in NCHFSs” we introduced exponential operational laws and some useful results in NCHFS. “Exponential aggregation operators” deals with exponential aggregation operators and their properties in NCHFS. In “Applications of neutrosophic cubic hesitant fuzzy weighted exponential aggregation operator to MADM and ME-MADM problems” we establish a MCDM method based on NCHFEA operators and use this method in MCDM problem.

## Preliminaries

### Definition 1

(Ref.^21^) A fuzzy set (FS) on a nonempty set *W* is a mapping $\Gamma :W\to [\mathrm{0,1}]$.

### Definition 2

(Ref.^16^) The cubic set (CS) on a nonempty set Z is defined by $\mu =\langle x;I\left(x\right),\delta (x)/x\in X\rangle$*,* where $I(x)$ is an IVFS on Z and $\delta (x)$ is an FS on Z*.*

### Definition 3

(Ref.^30^) A neutrosophic set associated with a crisp set S, is a set of the form $\mu =\langle e;{\xi }_{T}\left(e\right),{\xi }_{I}\left(e\right),{\xi }_{F}\left(e\right)/e\in S\rangle$ where ${\xi }_{T},{\xi }_{I},{\xi }_{F}:S\to [\mathrm{0,1}]$ respectively called a truth membership function, a non-membership function and a false membership function.

### Definition 4

(Ref.^17^) A neutrosophic cubic set in a nonempty set E is defined as a pair $(B,\mu )$ where $B=\langle x;{B}_{T}\left(e\right),{B}_{I}\left(e\right),{B}_{F}\left(e\right)/e\in E\rangle$ is an INS and $\mu =\langle x;{\mu }_{T}\left(e\right),{\mu }_{I}\left(e\right),{\mu }_{F}\left(e\right)/e\in X\rangle$ is a NS.

### Definition 5

(Ref.^5^) A neutrosophic hesitant fuzzy set a nonempty set E is described as $\mu =\langle x;{\mu }_{T}\left(e\right),{\mu }_{I}\left(e\right),{\mu }_{F}\left(e\right)/e\in E\rangle$ where ${\mu }_{T}\left(e\right),{\mu }_{I}\left(e\right),{\mu }_{F}\left(e\right)$ are three HFSs such that ${\mu }_{T}\left(e\right)+{\mu }_{I}\left(e\right)+{\mu }_{F}\left(e\right)\le 3$.

### Definition 6

(Ref.^38^) The object $\zeta =\langle x;{\xi }_{T}\left(x\right),{\xi }_{I}\left(x\right),{\xi }_{F}\left(x\right)/x\in X\rangle$, s called an INHFS on *X,* where ${\xi }_{T}\left(x\right),{\xi }_{I}\left(x\right),{\xi }_{F}\left(x\right)$ are IHFSs.

Zhu et al. proposed the following *β*-normalization method to enlarge a hesitant fuzzy element, which is a useful technique in case of different cardinalities.

### Definition 7

(Ref.^1^) Let ${m}^{+}$ and ${m}^{-}$ be the maximum and minimum elements of a hesitant fuzzy set H and $\zeta (0\le \zeta \le 1)$ an optimized parameter. We call $m=\zeta {m}^{+}+(1-\zeta ){m}^{-}$ an added element.

### Definition 8

(Ref.^40^) Let $A=\langle x,{T}_{x},{T}_{x},{F}_{x}\rangle$ be a SVNS. Then exponential laws in A are defined by

$$ \lambda^{A} = \left\{ \begin{array}{l} \left\langle {x,(\lambda )^{{1 - T_{x} }} ,1 - (\lambda )^{{I_{x} }} ,1 - (\lambda )^{{F_{x} }} } \right\rangle ;\lambda \in (0,1) \\ \left\langle {x,\left( {\frac{1}{\lambda }} \right)^{{1 - T_{x} }} ,1 - \left( {\frac{1}{\lambda }} \right)^{{I_{x} }} ,1 - \left( {\frac{1}{\lambda }} \right)^{{F_{x} }} } \right\rangle ;\lambda \ge 1 \end{array} \right.. $$

### Definition 9

(Ref.^52^) Let $A=\langle x,\left[{A}_{T}^{L}(x),{A}_{T}^{U}(x)\right],\left[{A}_{I}^{L}(x),{A}_{I}^{U}(x)\right],\left[{A}_{F}^{L}(x),{A}_{F}^{U}(x)\right]\rangle$ be an IVNS. Then exponential laws in A are defined by

$$ \lambda^{A} = \left\{ \begin{gathered} \left\langle {x,\left[ {(\lambda )^{{1 - A_{{{{{T} }} }}^{L} (x)}} ,(\lambda )^{{1 - A_{{{{{T} }} }}^{U} (x)}} } \right],\left[ {1 - (\lambda )^{{A_{{{{{I} }} }}^{L} (x)}} ,1 - (\lambda )^{{A_{{{{{{{I} }} }} }}^{U} (x)}} } \right],\left[ {1 - (\lambda )^{{A_{{{{{F} }} }}^{L} (x)}} ,\,1 - \left( \lambda \right)^{{A_{{{{{F} }} }}^{U} (x)}} } \right]} \right\rangle ;\lambda \in (0,1) \\ \left\langle {x,\left[ {\left( {\frac{1}{\lambda }} \right)^{{1 - A_{{{{{T} }} }}^{L} (x)}} ,\left( {\frac{1}{\lambda }} \right)^{{1 - A_{{{{{T} }} }}^{U} (x)}} } \right],\left[ {1 - \left( {\frac{1}{\lambda }} \right)^{{A_{{{{{I} }} }}^{L} (x)}} ,1 - \left( {\frac{1}{\lambda }} \right)^{{A_{{{I} }}^{U} (x)}} } \right],\left[ {1 - \left( {\frac{1}{\lambda }} \right)^{{A_{{{{{F} }} }}^{L} (x)}} ,\,1 - \left( {\frac{1}{\lambda }} \right)^{{A_{{{{{F} }} }}^{U} (x)}} } \right]} \right\rangle ;\lambda \ge 1 \\ \end{gathered} \right.. $$

## Operational laws in neutrosophic cubic hesitant fuzzy set

In this section operational laws on NCHFS are defined. These operational laws will help to define the proposed aggregations operators.

### Definition 10

Let X be a nonempty set. A neutrosophic cubic hesitant fuzzy set in X is a pair $\beta = \left\langle {B,\,\mu } \right\rangle$ where $B = \left\{ {\left\langle {x;\,B_{T} (x),\,B_{I} (x),\,B_{F} (x)} \right\rangle /x \in X} \right\}$ is an interval-valued neutrosophic hesitant set in X and $\mu = \left\{ {\left\langle {x;\,\vartheta_{T} (x),\,\vartheta_{I} (x),\,\vartheta_{F} (x)} \right\rangle /x \in X} \right\}$ is a neutrosophic hesitant set in X.

Furthermore $A_{T} = \left\{ {[A_{{j_{T} }}^{L} ,\,A_{{j_{T} }}^{U} ];\,j = 1,...,\,l} \right\},\,A_{I} = \left\{ {[A_{{j_{I} }}^{L} ,\,A_{{j_{I} }}^{U} ];\,j = 1,...,\,m} \right\},\,A_{F} = \left\{ {[A_{{j_{F} }}^{L} ,\,A_{{j_{F} }}^{U} ];\,j = 1,...,\,n} \right\}$ are some interval values in unit interval [0,1] and $\mu_{T} = \left\{ {\mu_{{j_{T} }} ;\,j = 1,...,\,p} \right\},\,\mu_{I} = \left\{ {\mu_{{j_{I} }} ;\,j = 1,...,\,q} \right\},\,\mu_{F} = \left\{ {\mu_{{j_{F} }} ;\,j = 1,...,\,r} \right\}$ are some values in unit interval [0,1].

### Example 1

Let $X = \left\{ {x,\,y,\,z} \right\}$ The pair $\alpha = \left\langle {A,\,\lambda } \right\rangle$ with

$$ A_{T} (x) = \left\{ {[0.1,\,0.5],[0.2,\,0.7]} \right\},\,\lambda_{T} (x) = \{ 0.3,\,0.5,0.7\} ,A_{I} (x) = \left\{ {[0.2,\,0.4],[0.3,\,0.6]} \right\},\,\lambda_{T} (x) = \{ 0.1,\,0.4,0.7\} ,A_{F} (x) = \left\{ {[0.1,\,0.4],[0,\,0.3],[0.6,0.8]} \right\},\,\lambda_{F} (x) = \{ 0.4,\,0.6\} $$

$$ A_{T} (y) = \left\{ {[0.1,\,0.5],[0.2,\,0.7]} \right\},\,\lambda_{T} (y) = \{ 0.3,\,0.5\} ,A_{I} (y) = \left\{ {[0.2,\,0.3],[0.1,\,0.6]} \right\},\,\lambda_{T} (y) = \{ 0.7,\,0.8\} ,A_{F} (y) = \left\{ {[0.1,\,0.4],[0,\,0.3]} \right\},\,\lambda_{F} (y) = \{ 0.4,\,0.6\} $$

$$ A_{T} (z) = \left\{ {[0.1,\,0.5],[0.2,\,0.7]} \right\},\,\lambda_{T} (z) = \{ 0.3,\,0.5\} ,A_{I} (z) = \left\{ {[0.2,\,0.3],[0.1,\,0.6]} \right\},\,\lambda_{I} (z) = \{ 0.7,\,0.8\} ,A_{F} (z) = \left\{ {[0.1,\,0.4],[0,\,0.3]} \right\},\,\lambda_{F} (z) = \{ 0.4,\,0.6\} $$

is a NCHFS.

### Definition 11

The sum and product of two NCHFSs $\alpha = \left\langle {A,\,\lambda } \right\rangle ,\,\beta = \left\langle {B,\,\mu } \right\rangle$ is defined as

$$ \alpha \oplus \beta = \left\langle \begin{gathered} x,\left\{ {\left[ {A_{{j_{{{T} }} }}^{L} + B_{{j_{{{T} }} }}^{L} - A_{{j_{{{T} }} }}^{L} B_{{j_{{{T} }} }}^{L} ,\,A_{{j_{{{T} }} }}^{U} + B_{{j_{T} }}^{U} - A_{{j_{T} }}^{U} B_{{j_{T} }}^{U} } \right]} \right\},\left\{ {\left[ {A_{{j_{I} }}^{L} + B_{{j_{I} }}^{L} - A_{{j_{I} }}^{L} B_{{j_{I} }}^{L} ,\,A_{{j_{{{I} }} }}^{U} + B_{{j_{I} }}^{U} - A_{{j_{I} }}^{U} B_{{j_{I} }}^{U} } \right]} \right\},\left\{ {[A_{{j_{F} }}^{L} B_{{j_{F} }}^{L} ,\,A_{{j_{F} }}^{U} B_{{j_{F} }}^{U} ]} \right\}, \\ \left\{ {\lambda_{{j_{T} }} + \mu_{{j_{T} }} - \lambda_{{j_{T} }} \mu_{{j_{T} }} } \right\},\left\{ {\lambda_{{j_{I} }} + \mu_{{j_{I} }} - \lambda_{{j_{I} }} \mu_{{j_{I} }} } \right\},\left\{ {\lambda_{{j_{F} }} } \right\} \\ \end{gathered} \right\rangle , $$

$$ \alpha \otimes \beta = \left\langle \begin{gathered} x,\left\{ {[A_{{j_{{{T} }} }}^{L} B_{{j_{T} }}^{L} ,\,A_{{j_{T} }}^{U} B_{{j_{T} }}^{U} ]} \right\},\left\{ {[A_{{j_{I} }}^{L} B_{{j_{I} }}^{L} ,\,A_{{j_{I} }}^{U} B_{{j_{I} }}^{U} ]} \right\},\left\{ {[A_{{j_{F} }}^{L} + B_{{j_{F} }}^{L} - A_{{j_{F} }}^{L} B_{{j_{F} }}^{L} ,\,A_{{j_{F} }}^{U} + B_{{j_{F} }}^{U} - A_{{j_{F} }}^{U} B_{{j_{F} }}^{U} ]} \right\}, \\ \left\{ {\lambda_{{j_{T} }} \mu_{{j_{T} }} } \right\},\left\{ {\lambda_{{j_{I} }} \mu_{{j_{I} }} } \right\},\left\{ {\lambda_{{j_{F} }} + \mu_{{j_{F} }} - \lambda_{{j_{F} }} \mu_{{j_{F} }} } \right\} \\ \end{gathered} \right\rangle . $$

Moreover the $\beta$-normalization is used in case of different cardinalities.

### Definition 12

The scalar multiplication of a scalar *q* with a NCHFS $\alpha = \left\langle {A,\,\lambda } \right\rangle$ is defined by

$$ q\alpha = \left\langle \begin{gathered} \left\{ {\left[ {1 - \left( {1 - A_{{j_{{{T} }} }}^{L} } \right)^{q} ,\,1 - \left( {1 - A_{{j_{T} }}^{U} } \right)^{q} } \right]} \right\},\left\{ {\left[ {1 - \left( {1 - A_{{j_{I} }}^{L} } \right)^{q} ,\,1 - \left( {1 - A_{{j_{I} }}^{U} } \right)^{q} } \right]} \right\},\left\{ {\left[ {\left( {A_{{j_{F} }}^{L} } \right)^{q} ,\left( {A_{{j_{F} }}^{U} } \right)^{q} } \right]} \right\}, \\ \left\{ {1 - \left( {1 - \lambda_{{j_{T} }} } \right)^{q} } \right\},\left\{ {1 - \left( {1 - \lambda_{{j_{I} }} } \right)^{q} } \right\},\left\{ {\left( {\lambda_{{j_{F} }} } \right)^{q} } \right\} \\ \end{gathered} \right\rangle . $$

### Theorem 1

*For NCHFS* $\alpha = \left\langle {A,\,\lambda } \right\rangle$* and a scalar* *q, we have*

$$ \alpha^{q} = \left\langle \begin{gathered} x,\left\{ {\left[ {\left( {A_{{p_{{{T} }} }}^{L} } \right)^{q} ,\left( {A_{{p_{T} }}^{U} } \right)^{q} } \right]} \right\},\left\{ {\left[ {\left( {A_{{p_{I} }}^{L} } \right)^{q} ,\left( {A_{{p_{I} }}^{U} } \right)} \right]^{q} } \right\},\left\{ {\left[ {1 - \left( {1 - A_{{p_{F} }}^{L} } \right)^{q} ,\,1 - \left( {1 - A_{{p_{F} }}^{U} } \right)^{q} } \right]} \right\}, \\ \left\{ {\left( {\lambda_{{p_{T} }} } \right)^{q} } \right\},\left\{ {\left( {\lambda_{{p_{I} }} } \right)^{q} } \right\},\left\{ {1 - \left( {1 - \lambda_{{p_{F} }} } \right)^{q} } \right\} \\ \end{gathered} \right\rangle $$

*where* $\alpha^{q} = \alpha \otimes \alpha \otimes \cdots \otimes \alpha (q{\text{ - times}}).$* moreover* $\alpha^{q}$ *is a neutrosophic cubic hesitant fuzzy value for every positive value of q.*

### Definition 13

The score, accuracy, and certainty of a NCHF value $\alpha = \left\langle {A,\,\lambda } \right\rangle$ where $A = \left( {A_{T} ,\,A_{I} ,\,A_{F} } \right),A_{T} = \left\{ {[A_{{j_{T} }}^{L} ,\,A_{{j_{T} }}^{U} ];\,j = 1, \ldots ,\,l} \right\},\,A_{I} = \left\{ {[A_{{j_{I} }}^{L} ,\,A_{{j_{I} }}^{U} ];\,j = 1, \ldots ,\,m} \right\},A_{F} = \left\{ {[A_{{j_{F} }}^{L} ,\,A_{{j_{F} }}^{U} ];\,j = 1, \ldots ,\,n} \right\}$ and $\lambda = (\lambda_{T} ,\,\lambda_{I} ,\,\lambda_{F} ),\lambda_{T} = \left\{ {\lambda_{{j_{T} }} ;\,j = 1, \ldots ,\,p} \right\},\,\lambda_{I} = \left\{ {\lambda_{{j_{I} }} ;\,j = 1, \ldots ,\,q} \right\},\lambda_{F} = \left\{ {\lambda_{{j_{F} }} ;\,j = 1, \ldots ,\,r} \right\}$ are defined as:

$$ S(\alpha ) = \frac{1}{2}\left\{ \begin{gathered} \frac{1}{6}\left( {\frac{1}{l}\mathop \sum \limits_{j = 1}^{l} \left( {A_{{j_{T} }}^{U} + A_{{j_{F} }}^{U} } \right) + \frac{1}{m}\mathop \sum \limits_{j = 1}^{m} \left( {A_{{j_{I} }}^{U} + A_{{j_{I} }}^{U} } \right) + \frac{1}{n}\mathop \sum \limits_{j = 1}^{n} \left( {2 - \left( {A_{{j_{T} }}^{L} + A_{{j_{F} }}^{L} } \right)} \right)} \right) \\ + \frac{1}{3}\left( {\frac{1}{p}\mathop \sum \limits_{j = 1}^{p} \lambda_{{j_{T} }} + \frac{1}{q}\mathop \sum \limits_{j = 1}^{q} \lambda_{{j_{I} }} + \frac{1}{r}\mathop \sum \limits_{j = 1}^{r} \left( {1 - \lambda_{{j_{F} }} } \right)} \right) \\ \end{gathered} \right\} $$

$$ H(\alpha ) = \tfrac{1}{3}\left\{ {\tfrac{1}{l}\mathop \sum \limits_{j = 1}^{l} \left( {A_{{j_{T} }}^{L} + A_{{j_{T} }}^{U} } \right) - \tfrac{1}{n}\mathop \sum \limits_{j = 1}^{n} \left( {A_{{j_{F} }}^{U} + A_{{j_{F} }}^{L} } \right) + \tfrac{1}{p}\mathop \sum \limits_{j = 1}^{p} \lambda_{{j_{T} }} - \tfrac{1}{r}\mathop \sum \limits_{j = 1}^{r} \lambda_{{j_{F} }} } \right\}, $$

$$ C(\alpha ) = \tfrac{1}{3}\left\{ {\tfrac{1}{l}\mathop \sum \limits_{j = 1}^{l} \left( {A_{{j_{T} }}^{L} + A_{{j_{T} }}^{U} } \right) + \tfrac{1}{p}\mathop \sum \limits_{j = 1}^{p} \lambda_{{j_{T} }} } \right\}. $$

### Remark

(i) It is evident from the above definition that for any NCHF value $\alpha = \left\langle {A,\,\lambda } \right\rangle$, $S(\alpha ) \in [0,1]$, $H(\alpha ) \in [ - 1,1]$ and $C(\alpha ) \in [0,1]$.

(ii) If $\Omega =\left(\left\{\left[\mathrm{1,1}\right]\right\},\left\{[\mathrm{1,1}]\right\},\left\{[\mathrm{0,0}]\right\},\left\{1\right\},\left\{1\right\},\left\{0\right\}\right)$ and $\Psi =\left(\left\{\left[\mathrm{0,0}\right]\right\},\left\{[\mathrm{0,0}]\right\},\left\{[\mathrm{1,1}]\right\},\left\{0\right\},\left\{0\right\},\left\{1\right\}\right)$ are respectively the maximum and minimum ideal NCHF values. Then $S(\Omega ) = 1,S(\Psi ) = 0$, $H(\Omega ) = 1,H(\Psi ) = - 1$, $C(\Omega ) = 1,C(\Psi ) = 0$.

If $\alpha = \left\langle {\left\{ {[0.1,\,0.5],[0.2,\,0.7]} \right\},\left\{ {[0.2,\,0.3],[0.1,\,0.6]} \right\},\left\{ {[0.1,\,0.4],[0,\,0.3]} \right\},\{ 0.1,\,0.2\} ,\{ 0.3,\,0.5\} ,\{ 0.4,\,0.8\} } \right\rangle ,$ and $\beta = \left\langle {\left\{ {[0.4,\,0.5],[0.3,\,0.4]} \right\},\left\{ {[0.1,\,0.3],[0.2,\,0.5]} \right\},\left\{ {[0.1,\,0.4],[0.7,\,0.8]} \right\},\{ 0.3,\,0.5\} ,\{ 0.7,\,0.8\} ,\{ 0.4,\,0.6\} } \right\rangle ,$ then $S(\alpha ) = 0.370833,\,S(\beta ) = 0.545833,\,H(\alpha ) = - 0.03333,\,H(\beta ) = - 0.1,C(\alpha ) = 0.3,C(\beta ) = 0.4 .$

**Figure 1:**
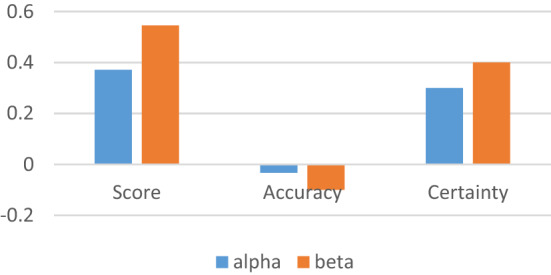
Score, accuracy, and certainty.

Figure 1 provides the graphical interpretation of score, accuracy and cetainty functions of NCHF values. The value with greater score is defined to be greater than other.

### Definition 14

Let $\alpha =\langle A,\lambda \rangle ,\beta =\langle B,\mu \rangle$ are two NCHFSs. We say that $\alpha >\beta$ if $S(\alpha )>S(\beta )$. If $S\left(\alpha \right)=S(\beta )$, then $\alpha >\beta$ if $A(\alpha )>A(\beta )$. If $A\left(\alpha \right)=A(\beta )$, then $\alpha >\beta$ if $C(\alpha )>C(\beta )$. If $S\left(\alpha \right)=S\left(\beta \right), A\left(\alpha \right)=A\left(\beta \right),C(\alpha )>C(\beta )$, then $\alpha =\beta$.

In the next section we define exponential operational laws in neutrosophic cubic hesitant fuzzy set and prove some elegant results.

## Exponential operational laws in NCHFSs

### Definition 15

For NCHFS $\alpha = \left\langle {A,\,\lambda } \right\rangle$ and a scalar $q>0$, we define

$$ q^{\alpha } = \left\{ \begin{gathered} \left\langle \begin{gathered} x,\left\{ {\left[ {(q)^{{1 - A_{{j_{{{T} }} }}^{L} }} ,(q)^{{1 - A_{{j_{{{T} }} }}^{U} }} } \right]} \right\},\left\{ {\left[ {(q)^{{1 - A_{{j_{{{I} }} }}^{L} }} ,(q)^{{1 - A_{{{{j_{{{I} }} }} }}^{U} }} } \right]} \right\},\left\{ {\left[ {1 - (q)^{{A_{{j_{{{F} }} }}^{L} }} ,\,1 - \left( q \right)^{{A_{{j_{{{F} }} }}^{U} }} } \right]} \right\}, \\ \left\{ {\left( q \right)^{{1 - \lambda_{{j_{{{T} }} }} }} } \right\},\left\{ {\left( q \right)^{{1 - \lambda_{{j_{{{I} }} }} }} } \right\},\left\{ {\left( {1 - q} \right)^{{\lambda_{{j_{{{F} }} }} }} } \right\} \\ \end{gathered} \right\rangle ;q \in (0,1) \\ \left\langle \begin{gathered} x,\left\{ {\left[ {\left( \frac{1}{q} \right)^{{1 - A_{{j_{{{T} }} }}^{L} }} ,\left( \frac{1}{q} \right)^{{1 - A_{{j_{{{T} }} }}^{U} }} } \right]} \right\},\left\{ {\left[ {\left( \frac{1}{q} \right)^{{1 - A_{{j_{{{I} }} }}^{L} }} ,\left( \frac{1}{q} \right)^{{1 - A_{{{{j_{{{I} }} }} }}^{U} }} } \right]} \right\},\left\{ {\left[ {1 - \left( \frac{1}{q} \right)^{{A_{{j_{{{F} }} }}^{L} }} ,\,1 - \left( \frac{1}{q} \right)^{{A_{{j_{{{F} }} }}^{U} }} } \right]} \right\}, \\ \left\{ {\left( \frac{1}{q} \right)^{{1 - \lambda_{{j_{{{T} }} }} }} } \right\},\left\{ {\left( \frac{1}{q} \right)^{{1 - \lambda_{{j_{{{I} }} }} }} } \right\},\left\{ {1 - \left( \frac{1}{q} \right)^{{\lambda_{{j_{{{F} }} }} }} } \right\} \\ \end{gathered} \right\rangle ;q \ge 1 \\ \end{gathered} \right.. $$

If $$\alpha >\beta $$, then $${q}^{\alpha }>{q}^{\beta }$$.

### Example 2

$\alpha = \left\langle {\left\{ {[0.1,\,0.5],[0.2,\,0.7]} \right\},\left\{ {[0.2,\,0.3],[0.1,\,0.6]} \right\},\left\{ {[0.1,\,0.4],[0.0,\,0.3]} \right\},\{ 0.1,\,0.2\} ,\{ 0.3,\,0.5\} ,\{ 0.4,\,0.8\} } \right\rangle$, then using above definition with *q* = *0.5* we have

$$ q\alpha = \left\langle \begin{gathered} \left\{ {\left[ {0.535887,\,0.707107} \right],\left[ {0.574349,\,0.812252} \right]} \right\},\left\{ {\left[ {0.574349,\,0.615572} \right],\left[ {0.535887,\,0.757858} \right]} \right\}, \\ \left\{ {\left[ {0.066967,\,0.242142} \right],\left[ {0,\,0.187748} \right]} \right\},\{ 0.535887,\,0.574349\} , \\ \{ 0.615572,\,0.707107\} ,\{ 0.242142,\,0.425651\} \\ \end{gathered} \right\rangle $$

### Theorem 2

*For a NCHFN* $\alpha = \left\langle {A,\,\lambda } \right\rangle$* and a scalar* $q>0$, $q^{\alpha }$* is a NCHFN.*

### Theorem 3

*For two NCHFNs* $\alpha = \left\langle {A,\,\lambda } \right\rangle ,\,\beta = \left\langle {B,\,\mu } \right\rangle$* and a scalar* $q\in (\mathrm{0,1})$*, we have.*

 (i) $$q^{\alpha } \oplus q^{\beta } = q^{\beta } \oplus q^{\alpha } ,$$ (ii) $$q^{\alpha } \otimes q^{\beta } = q^{\beta } \otimes q^{\alpha }$$.

**Proof**

**(i)**

$$ \begin{gathered} q^{\alpha } \oplus q^{\beta } = \left\langle \begin{gathered} \left\{ {\left[ {(q)^{{1 - A_{{j_{{{T} }} }}^{L} }} ,(q)^{{1 - A_{{j_{{{T} }} }}^{U} }} } \right]} \right\},\left\{ {\left[ {(q)^{{1 - A_{{j_{{{I} }} }}^{L} }} ,(q)^{{1 - A_{{{{j_{{{I} }} }} }}^{U} }} } \right]} \right\}, \\ \left\{ {\left[ {1 - (q)^{{A_{{j_{{{F} }} }}^{L} }} ,\,1 - \left( q \right)^{{A_{{j_{{{F} }} }}^{U} }} } \right]} \right\}, \\ \left\{ {\left( q \right)^{{1 - \lambda_{{j_{{{T} }} }} }} } \right\},\left\{ {\left( q \right)^{{1 - \lambda_{{j_{{{I} }} }} }} } \right\},\left\{ {1 - \left( q \right)^{{\lambda_{{j_{{{F} }} }} }} } \right\} \\ \end{gathered} \right\rangle \oplus \left\langle \begin{gathered} \left\{ {\left[ {(q)^{{1 - B_{{j_{{{T} }} }}^{L} }} ,(q)^{{1 - B_{{j_{{{T} }} }}^{U} }} } \right]} \right\},\left\{ {\left[ {(q)^{{1 - B_{{j_{{{I} }} }}^{L} }} ,(q)^{{1 - B_{{{{j_{{{I} }} }} }}^{U} }} } \right]} \right\}, \\ \left\{ {\left[ {1 - (q)^{{B_{{j_{{{F} }} }}^{L} }} ,\,1 - \left( q \right)^{{B_{{j_{{{F} }} }}^{U} }} } \right]} \right\}, \\ \left\{ {\left( q \right)^{{1 - \mu_{{j_{{{T} }} }} }} } \right\},\left\{ {\left( q \right)^{{1 - \mu_{{j_{{{I} }} }} }} } \right\},\left\{ {1 - \left( q \right)^{{\mu_{{j_{{{F} }} }} }} } \right\} \\ \end{gathered} \right\rangle \hfill \\ = \left\langle \begin{gathered} \left\{ {\left[ {(q)^{{1 - A_{{j_{{{T} }} }}^{L} }} + (q)^{{1 - B_{{j_{{{T} }} }}^{L} }} - (q)^{{1 - A_{{j_{{{T} }} }}^{L} }} (q)^{{1 - B_{{j_{{{T} }} }}^{L} }} ,(q)^{{1 - A_{{j_{{{T} }} }}^{U} }} + (q)^{{1 - B_{{j_{{{T} }} }}^{U} }} - (q)^{{1 - A_{{j_{{{T} }} }}^{U} }} (q)^{{1 - B_{{j_{{{T} }} }}^{U} }} } \right]} \right\}, \\ \left\{ {\left[ {(q)^{{1 - A_{{j_{{{I} }} }}^{L} }} + (q)^{{1 - B_{{j_{{{I} }} }}^{L} }} - (q)^{{1 - A_{{j_{{{I} }} }}^{L} }} (q)^{{1 - B_{{j_{{{I} }} }}^{L} }} ,(q)^{{1 - A_{{{{j_{{{I} }} }} }}^{U} }} + (q)^{{1 - B_{{{{j_{{{I} }} }} }}^{U} }} - (q)^{{1 - A_{{{{j_{{{I} }} }} }}^{U} }} (q)^{{1 - B_{{{{j_{{{I} }} }} }}^{U} }} } \right]} \right\} \\ ,\left\{ {\left[ {\left( {1 - (q)^{{A_{{j_{{{F} }} }}^{L} }} } \right)\left( {1 - (q)^{{B_{{j_{{{F} }} }}^{L} }} } \right),\,\left( {1 - \left( q \right)^{{A_{{j_{{{F} }} }}^{U} }} } \right)\left( {1 - \left( q \right)^{{B_{{j_{{{F} }} }}^{U} }} } \right)} \right]} \right\}, \\ \left\{ {\left( q \right)^{{1 - \lambda_{{j_{{{T} }} }} }} + \left( q \right)^{{1 - \mu_{{j_{{{T} }} }} }} - \left( q \right)^{{1 - \lambda_{{j_{{{T} }} }} }} \left( q \right)^{{1 - \mu_{{j_{{{T} }} }} }} } \right\},\left\{ {\left( q \right)^{{1 - \lambda_{{j_{{{I} }} }} }} + \left( q \right)^{{1 - \mu_{{j_{{{I} }} }} }} - \left( q \right)^{{1 - \lambda_{{j_{{{I} }} }} }} \left( q \right)^{{1 - \mu_{{j_{{{I} }} }} }} } \right\},\left\{ {\left( {1 - q} \right)^{{\lambda_{{j_{{{F} }} }} }} \left( {1 - q} \right)^{{\mu_{{j_{{{F} }} }} }} } \right\} \\ \end{gathered} \right\rangle \hfill \\ = \left\langle \begin{gathered} \left\{ {\left[ {(q)^{{1 - B_{{j_{{{T} }} }}^{L} }} + (q)^{{1 - A_{{j_{{{T} }} }}^{L} }} - (q)^{{1 - B_{{j_{{{T} }} }}^{L} }} (q)^{{1 - A_{{j_{{{T} }} }}^{L} }} ,(q)^{{1 - B_{{j_{{{T} }} }}^{U} }} + (q)^{{1 - A_{{j_{{{T} }} }}^{U} }} - (q)^{{1 - B_{{j_{{{T} }} }}^{U} }} (q)^{{1 - A_{{j_{{{T} }} }}^{U} }} } \right]} \right\}, \\ \left\{ {\left[ {(q)^{{1 - B_{{j_{{{I} }} }}^{L} }} + (q)^{{1 - A_{{j_{{{I} }} }}^{L} }} - (q)^{{1 - B_{{j_{{{I} }} }}^{L} }} (q)^{{1 - A_{{j_{{{I} }} }}^{L} }} ,(q)^{{1 - B_{{{{j_{{{I} }} }} }}^{U} }} + (q)^{{1 - A_{{{{j_{{{I} }} }} }}^{U} }} - (q)^{{1 - B_{{{{j_{{{I} }} }} }}^{U} }} (q)^{{1 - A_{{{{j_{{{I} }} }} }}^{U} }} } \right]} \right\} \\ ,\left\{ {\left[ {\left( {1 - (q)^{{B_{{j_{{{F} }} }}^{L} }} } \right)\left( {1 - (q)^{{A_{{j_{{{F} }} }}^{L} }} } \right),\,\left( {1 - \left( q \right)^{{B_{{j_{{{F} }} }}^{U} }} } \right)\left( {1 - \left( q \right)^{{A_{{j_{{{F} }} }}^{U} }} } \right)} \right]} \right\}, \\ \left\{ {\left( q \right)^{{1 - \mu_{{j_{{{T} }} }} }} + \left( q \right)^{{1 - \lambda_{{j_{{{T} }} }} }} - \left( q \right)^{{1 - \mu_{{j_{{{T} }} }} }} \left( q \right)^{{1 - \lambda_{{j_{{{T} }} }} }} } \right\},\left\{ {\left( q \right)^{{1 - \mu_{{j_{{{I} }} }} }} + \left( q \right)^{{1 - \lambda_{{j_{{{I} }} }} }} - \left( q \right)^{{1 - \mu_{{j_{{{I} }} }} }} \left( q \right)^{{1 - \lambda_{{j_{{{I} }} }} }} } \right\},\left\{ {\left( {1 - q} \right)^{{\mu_{{j_{{{F} }} }} }} \left( {1 - q} \right)^{{\lambda_{{j_{{{F} }} }} }} } \right\} \\ \end{gathered} \right\rangle = q^{\beta } \oplus q^{\alpha } . \hfill \\ \end{gathered} $$

(ii)

$$ \begin{gathered} q^{\alpha } \otimes q^{\beta } = \left\langle \begin{gathered} \left\{ {\left[ {(q)^{{1 - A_{{j_{{{T} }} }}^{L} }} ,(q)^{{1 - A_{{j_{{{T} }} }}^{U} }} } \right]} \right\},\left\{ {\left[ {(q)^{{1 - A_{{j_{{{I} }} }}^{L} }} ,(q)^{{1 - A_{{{{j_{{{I} }} }} }}^{U} }} } \right]} \right\}, \\ \left\{ {\left[ {1 - (q)^{{A_{{j_{{{F} }} }}^{L} }} ,\,1 - \left( q \right)^{{A_{{j_{{{F} }} }}^{U} }} } \right]} \right\}, \\ \left\{ {\left( q \right)^{{1 - \lambda_{{j_{{{T} }} }} }} } \right\},\left\{ {\left( q \right)^{{1 - \lambda_{{j_{{{I} }} }} }} } \right\},\left\{ {1 - \left( q \right)^{{\lambda_{{j_{{{F} }} }} }} } \right\} \\ \end{gathered} \right\rangle \otimes \left\langle \begin{gathered} \left\{ {\left[ {(q)^{{1 - B_{{j_{{{T} }} }}^{L} }} ,(q)^{{1 - B_{{j_{{{T} }} }}^{U} }} } \right]} \right\},\left\{ {\left[ {(q)^{{1 - B_{{j_{{{I} }} }}^{L} }} ,(q)^{{1 - B_{{{{j_{{{I} }} }} }}^{U} }} } \right]} \right\}, \\ \left\{ {\left[ {1 - (q)^{{B_{{j_{{{F} }} }}^{L} }} ,\,1 - \left( q \right)^{{B_{{j_{{{F} }} }}^{U} }} } \right]} \right\}, \\ \left\{ {\left( q \right)^{{1 - \mu_{{j_{{{T} }} }} }} } \right\},\left\{ {\left( q \right)^{{1 - \mu_{{j_{{{I} }} }} }} } \right\},\left\{ {1 - \left( q \right)^{{\mu_{{j_{{{F} }} }} }} } \right\} \\ \end{gathered} \right\rangle \hfill \\ = \left\langle \begin{gathered} \left\{ {\left[ {(q)^{{1 - A_{{j_{{{T} }} }}^{L} }} (q)^{{1 - B_{{j_{{{T} }} }}^{L} }} ,(q)^{{1 - A_{{j_{{{T} }} }}^{U} }} (q)^{{1 - B_{{j_{{{T} }} }}^{U} }} } \right]} \right\},\left\{ {\left[ {(q)^{{1 - A_{{j_{{{I} }} }}^{L} }} (q)^{{1 - B_{{j_{{{I} }} }}^{L} }} ,(q)^{{1 - A_{{{{j_{{{I} }} }} }}^{U} }} (q)^{{1 - B_{{{{j_{{{I} }} }} }}^{U} }} } \right]} \right\}, \\ \left\{ {\left[ \begin{gathered} 1 - (q)^{{A_{{j_{{{F} }} }}^{L} }} + 1 - (q)^{{B_{{j_{{{F} }} }}^{L} }} - \left( {1 - (q)^{{A_{{j_{{{F} }} }}^{L} }} } \right)\left( {1 - (q)^{{B_{{j_{{{F} }} }}^{L} }} } \right),\, \hfill \\ 1 - \left( q \right)^{{A_{{j_{{{F} }} }}^{U} }} + 1 - \left( q \right)^{{B_{{j_{{{F} }} }}^{U} }} + \left( {1 - \left( q \right)^{{A_{{j_{{{F} }} }}^{U} }} } \right)\left( {1 - \left( q \right)^{{B_{{j_{{{F} }} }}^{U} }} } \right) \hfill \\ \end{gathered} \right]} \right\}, \\ \left\{ {\left( q \right)^{{1 - \lambda_{{j_{{{T} }} }} }} \left( q \right)^{{1 - \mu_{{j_{{{T} }} }} }} } \right\},\left\{ {\left( q \right)^{{1 - \lambda_{{j_{{{I} }} }} }} \left( q \right)^{{1 - \mu_{{j_{{{I} }} }} }} } \right\}, \\ \left\{ {1 - (q)^{{\lambda_{{j_{{{F} }} }} }} + 1 - (q)^{{\mu_{{j_{{{F} }} }} }} - \left( {1 - (q)^{{\lambda_{{j_{{{F} }} }} }} } \right)\left( {1 - (q)^{{\mu_{{j_{{{F} }} }} }} } \right)} \right\} \\ \end{gathered} \right\rangle \hfill \\ = \left\langle \begin{gathered} \left\{ {\left[ {(q)^{{1 - B_{{j_{{{T} }} }}^{L} }} (q)^{{1 - A_{{j_{{{T} }} }}^{L} }} ,(q)^{{1 - B_{{j_{{{T} }} }}^{U} }} (q)^{{1 - A_{{j_{{{T} }} }}^{U} }} } \right]} \right\},\left\{ {\left[ {(q)^{{1 - B_{{j_{{{I} }} }}^{L} }} (q)^{{1 - A_{{j_{{{I} }} }}^{L} }} ,(q)^{{1 - B_{{{{j_{{{I} }} }} }}^{U} }} (q)^{{1 - A_{{{{j_{{{I} }} }} }}^{U} }} } \right]} \right\}, \\ \left\{ {\left[ \begin{gathered} 1 - (q)^{{B_{{j_{{{F} }} }}^{L} }} + 1 - (q)^{{A_{{j_{{{F} }} }}^{L} }} - \left( {1 - (q)^{{B_{{j_{{{F} }} }}^{L} }} } \right)\left( {1 - (q)^{{A_{{j_{{{F} }} }}^{L} }} } \right),\, \hfill \\ 1 - \left( q \right)^{{B_{{j_{{{F} }} }}^{U} }} + 1 - \left( q \right)^{{A_{{j_{{{F} }} }}^{U} }} + \left( {1 - \left( q \right)^{{B_{{j_{{{F} }} }}^{U} }} } \right)\left( {1 - \left( q \right)^{{A_{{j_{{{F} }} }}^{U} }} } \right) \hfill \\ \end{gathered} \right]} \right\}, \\ \left\{ {\left( q \right)^{{1 - \mu_{{j_{{{T} }} }} }} \left( q \right)^{{1 - \lambda_{{j_{{{T} }} }} }} } \right\},\left\{ {\left( q \right)^{{1 - \mu_{{j_{{{I} }} }} }} \left( q \right)^{{1 - \lambda_{{j_{{{I} }} }} }} } \right\}, \\ \left\{ {1 - (q)^{{\mu_{{j_{{{F} }} }} }} + 1 - (q)^{{\lambda_{{j_{{{F} }} }} }} - \left( {1 - (q)^{{\mu_{{j_{{{F} }} }} }} } \right)\left( {1 - (q)^{{\lambda_{{j_{{{F} }} }} }} } \right)} \right\} \\ \end{gathered} \right\rangle = q^{\beta } \otimes q^{\alpha } \hfill \\ \end{gathered} $$

### Theorem 4

*For three NCHFNs* $\alpha = \left\langle {A,\,\lambda } \right\rangle ,\,\beta = \left\langle {B,\,\mu } \right\rangle ,\gamma = \left\langle {C,\nu } \right\rangle$* and a scalar* $q\in (\mathrm{0,1})$*, we have.*

(i) $$\left( {q^{\alpha } \oplus q^{\beta } } \right) \oplus q^{\gamma } = q^{\beta } \oplus \left( {q^{\alpha } \oplus q^{\gamma } } \right) ,$$ (ii) $$\left( {q^{\alpha } \otimes q^{\beta } } \right) \otimes q^{\gamma } = q^{\beta } \otimes \left( {q^{\alpha } \otimes q^{\gamma } } \right)$$

**Proof: (i)**

$$ \begin{gathered} \left( {q^{\alpha } \oplus q^{\beta } } \right) \oplus q^{\gamma } = \left\langle \begin{gathered} \left\{ {\left[ \begin{gathered} \left( {\left( {(q)^{{1 - A_{{j_{{{T} }} }}^{L} }} + (q)^{{1 - B_{{j_{{{T} }} }}^{L} }} } \right) + (q)^{{1 - C_{{j_{{{T} }} }}^{L} }} } \right) - \left( {(q)^{{1 - A_{{j_{{{T} }} }}^{L} }} (q)^{{1 - B_{{j_{{{T} }} }}^{L} }} } \right)(q)^{{1 - C_{{j_{{{T} }} }}^{L} }} ,\, \hfill \\ \left( {\left( {(q)^{{1 - A_{{j_{{{T} }} }}^{U} }} + (q)^{{1 - B_{{j_{{{T} }} }}^{U} }} } \right) + (q)^{{1 - C_{{j_{{{T} }} }}^{U} }} } \right) - \left( {(q)^{{1 - A_{{j_{{{T} }} }}^{U} }} (q)^{{1 - B_{{j_{{{T} }} }}^{U} }} } \right)(q)^{{1 - C_{{j_{{{T} }} }}^{U} }} \hfill \\ \end{gathered} \right]} \right\}, \\ \left\{ {\left[ \begin{gathered} \left( {\left( {(q)^{{1 - A_{{j_{{{I} }} }}^{L} }} + (q)^{{1 - B_{{j_{{{I} }} }}^{L} }} } \right) + (q)^{{1 - C_{{j_{{{I} }} }}^{L} }} } \right) - \left( {(q)^{{1 - A_{{j_{{{I} }} }}^{L} }} (q)^{{1 - B_{{j_{{{I} }} }}^{L} }} } \right)(q)^{{1 - C_{{j_{{{I} }} }}^{L} }} ,\, \hfill \\ \left( {\left( {(q)^{{1 - A_{{{{j_{{{I} }} }} }}^{U} }} + (q)^{{1 - B_{{{{j_{{{I} }} }} }}^{U} }} } \right) + (q)^{{1 - C_{{{{j_{{{I} }} }} }}^{U} }} } \right) - \left( {(q)^{{1 - A_{{{{j_{{{I} }} }} }}^{U} }} (q)^{{1 - B_{{{{j_{{{I} }} }} }}^{U} }} } \right)(q)^{{1 - C_{{{{j_{{{I} }} }} }}^{U} }} \hfill \\ \end{gathered} \right]} \right\}, \\ \left\{ {\left[ \begin{gathered} \left( {\left( {1 - (q)^{{A_{{j_{{{F} }} }}^{L} }} } \right)\left( {1 - (q)^{{B_{{j_{{{F} }} }}^{L} }} } \right)} \right)\left( {1 - (q)^{{C_{{j_{{{F} }} }}^{L} }} } \right), \hfill \\ \left( {\left( {1 - \left( q \right)^{{A_{{j_{{{F} }} }}^{U} }} } \right)\left( {1 - \left( q \right)^{{B_{{j_{{{F} }} }}^{U} }} } \right)} \right)\left( {1 - \left( q \right)^{{C_{{j_{{{F} }} }}^{U} }} } \right) \hfill \\ \end{gathered} \right]} \right\}, \\ \left\{ {\left( {\left( {(q)^{{1 - \lambda_{{j_{{{T} }} }} }} + (q)^{{1 - \mu_{{j_{{{T} }} }} }} } \right) + (q)^{{1 - \nu_{{j_{{{T} }} }} }} } \right) - \left( {(q)^{{1 - \lambda_{{j_{{{T} }} }} }} (q)^{{1 - \mu_{{j_{{{T} }} }} }} } \right)(q)^{{1 - \nu_{{j_{{{T} }} }} }} } \right\}, \\ \left\{ {\left( {\left( {(q)^{{1 - \lambda_{{j_{{{I} }} }} }} + (q)^{{1 - \mu_{{j_{{{I} }} }} }} } \right) + (q)^{{1 - \nu_{{j_{{{I} }} }} }} } \right) - \left( {(q)^{{1 - \lambda_{{j_{{{I} }} }} }} (q)^{{1 - \mu_{{j_{{{I} }} }} }} } \right)(q)^{{1 - \nu_{{j_{{{I} }} }} }} } \right\}, \\ \left\{ {\left( {\left( {1 - \left( q \right)^{{\lambda_{{j_{{{F} }} }} }} } \right)\left( {1 - \left( q \right)^{{\mu_{{j_{{{F} }} }} }} } \right)} \right)\left( {1 - \left( q \right)^{{\nu_{{j_{F} }} }} } \right)} \right\} \\ \end{gathered} \right\rangle \hfill \\ = q^{\beta } \oplus \left( {q^{\alpha } \oplus q^{\gamma } } \right)\,\,\,\therefore \,\,\,\,{\text{by associativity of real numbers}}. \hfill \\ \end{gathered} $$

### Theorem 5

*For three NCHFNs* $\alpha = \left\langle {A,\,\lambda } \right\rangle ,\,\beta = \left\langle {B,\,\mu } \right\rangle ,\gamma = \left\langle {C,\nu } \right\rangle$*, a scalar *$q,{q}_{1}\in (\mathrm{0,1})$*, and* $k,{k}_{1},{k}_{2}>0$*, we have.*

(i) $$k\left( {q^{\alpha } \oplus q^{\beta } } \right) = kq^{\alpha } \oplus kq^{\beta }$$,
(ii) $$\left( {q^{\alpha } \otimes q^{\beta } } \right)^{k} = \left( {q^{\alpha } } \right)^{k} \otimes \left( {q^{\beta } } \right)^{k}$$,
(iii) $$\left( {k_{1} + k_{2} } \right)q^{\alpha } = k_{1} q^{\alpha } \oplus k_{2} q^{\alpha }$$,
(iv) $$\left( {q^{\alpha } } \right)^{{k_{1} }} \otimes \left( {q^{\alpha } } \right)^{{k_{2} }} = \left( {q^{\alpha } } \right)^{{k_{1} + k_{2} }}$$,
(v) $$q^{\alpha } \otimes q_{1}^{\alpha } = \left( {qq_{1} } \right)^{\alpha }$$

### Proof

(i)

$$ k\left( {{q^\alpha } \oplus {q^\beta }} \right) = k\left\langle \begin{array}{c}
\left\{ {\left[ {{{(q)}^{1 - A_{{j_{T}}}^L}} + {{(q)}^{1 - B_{{j_{T}}}^L}} - {{(q)}^{1 - A_{{j_{T}}}^L}}{{(q)}^{1 - B_{{j_{T}}}^L}},\,{{(q)}^{1 - A_{{j_{T}}}^U}} + {{(q)}^{1 - B_{{j_{T}}}^U}} - {{(q)}^{1 - A_{{j_{T}}}^U}}{{(q)}^{1 - B_{{j_{T}}}^U}}} \right]} \right\},\\
\left\{ {\left[ {{{(q)}^{1 - A_{{j_{I}}}^L}} + {{(q)}^{1 - B_{{j_{I}}}^L}} - {{(q)}^{1 - A_{{j_{I}}}^L}}{{(q)}^{1 - B_{{j_{I}}}^L}},\,{{(q)}^{1 - A_{{{j_{I}}}}^U}} + {{(q)}^{1 - B_{{{j_{I}}}}^U}} - {{(q)}^{1 - A_{{{j_{I}}}}^U}}{{(q)}^{1 - B_{{{j_{I}}}}^U}}} \right]} \right\},\\
\left\{ {\left[ {\left( {1 - {{(q)}^{A_{{j_{F}}}^L}}} \right)\left( {1 - {{(q)}^{B_{{j_{F}}}^L}}} \right),\,\left( {1 - {{\left( q \right)}^{A_{{j_{F}}}^U}}} \right)\left( {1 - {{\left( q \right)}^{B_{{j_{F}}}^U}}} \right)} \right]} \right\},\left\{ {{{(q)}^{1 - {\lambda _{{j_{T}}}}}} + {{(q)}^{1 - {\mu _{{j_{T}}}}}} - {{(q)}^{1 - {\lambda _{{j_{T}}}}}}{{(q)}^{1 - {\mu _{{j_{T}}}}}}} \right\},\\
\left\{ {{{(q)}^{1 - {\lambda _{{j_{I}}}}}} + {{(q)}^{1 - {\mu _{{j_{I}}}}}} - {{(q)}^{1 - {\lambda _{{j_{I}}}}}}{{(q)}^{1 - {\mu _{{j_{I}}}}}}} \right\},\left\{ {\left( {1 - {{\left( q \right)}^{{\lambda _{{j_{F}}}}}}} \right)\left( {1 - {{\left( q \right)}^{{\mu _{{j_{F}}}}}}} \right)} \right\}
\end{array} \right\rangle $$

$$ = \left\langle \begin{array}{c}
\left\{ {\left[ {1 - {{\left( {1 - \left( {{{(q)}^{1 - A_{{j_{T}}}^L}} + {{(q)}^{1 - B_{{j_{T}}}^L}} - {{(q)}^{1 - A_{{j_{T}}}^L}}{{(q)}^{1 - B_{{j_{T}}}^L}}} \right)} \right)}^k},\,1 - {{\left( {1 - \,\left( {{{(q)}^{1 - A_{{j_{T}}}^U}} + {{(q)}^{1 - B_{{j_{T}}}^U}} - {{(q)}^{1 - A_{{j_{T}}}^U}}{{(q)}^{1 - B_{{j_{T}}}^U}}} \right)} \right)}^k}} \right]} \right\},\\
\left\{ {\left[ {1 - {{\left( {1 - \left( {{{(q)}^{1 - A_{{j_{I}}}^L}} + {{(q)}^{1 - B_{{j_{I}}}^L}} - {{(q)}^{1 - A_{{j_{I}}}^L}}{{(q)}^{1 - B_{{j_{I}}}^L}}} \right)} \right)}^k},\,1 - {{\left( {1 - \left( {{{(q)}^{1 - A_{{{j_{I}}}}^U}} + {{(q)}^{1 - B_{{{j_{I}}}}^U}} - {{(q)}^{1 - A_{{{j_{I}}}}^U}}{{(q)}^{1 - B_{{{j_{I}}}}^U}}} \right)} \right)}^k}} \right]} \right\},\\
\left\{ {\left[ {{{\left( {\left( {1 - {{(q)}^{A_{{j_{F}}}^L}}} \right)\left( {1 - {{(q)}^{B_{{j_{F}}}^L}}} \right)} \right)}^k},{{\left( {\left( {1 - {{\left( q \right)}^{A_{{j_{F}}}^U}}} \right)\left( {1 - {{\left( q \right)}^{B_{{j_{F}}}^U}}} \right)} \right)}^k}} \right]} \right\},\\
\left\{ {1 - {{\left( {1 - \left( {{{(q)}^{1 - {\lambda _{{j_{T}}}}}} + {{(q)}^{1 - {\mu _{{j_{T}}}}}} - {{(q)}^{1 - {\lambda _{{j_{T}}}}}}{{(q)}^{1 - {\mu _{{j_{T}}}}}}} \right)} \right)}^k}} \right\},\\
\left\{ {1 - {{\left( {1 - \left( {{{(q)}^{1 - {\lambda _{{j_{I}}}}}} + {{(q)}^{1 - {\mu _{{j_{I}}}}}} - {{(q)}^{1 - {\lambda _{{j_{I}}}}}}{{(q)}^{1 - {\mu _{{j_{I}}}}}}} \right)} \right)}^k}} \right\},\left\{ {{{\left( {\left( {1 - {{\left( q \right)}^{{\lambda _{{j_{F}}}}}}} \right)\left( {1 - {{\left( q \right)}^{{\mu _{{j_{F}}}}}}} \right)} \right)}^k}} \right\}
\end{array} \right\rangle .
$$

$$k{q^\alpha } \oplus k{q^\beta } = \left\langle \begin{array}{c}
\left\{ {\left[ {1 - {{\left( {1 - {{(q)}^{1 - A_{{j_{T}}}^L}}} \right)}^k},\,1 - {{\left( {1 - {{(q)}^{1 - A_{{j_{T}}}^U}}} \right)}^k}} \right]} \right\},\\
\left\{ {\left[ {1 - {{\left( {1 - {{(q)}^{1 - A_{{j_{I}}}^L}}} \right)}^k},1 - {{\left( {1 - {{(q)}^{1 - A_{{{j_{I}}}}^U}}} \right)}^k}} \right]} \right\},\\
\left\{ {\left[ {{{\left( {1 - {{(q)}^{A_{{j_{F}}}^L}}} \right)}^k},{{\left( {1 - {{\left( q \right)}^{A_{{j_{F}}}^U}}} \right)}^k}} \right]} \right\},\\
\left\{ {1 - {{\left( {1 - {{(q)}^{1 - {\lambda _{{j_{T}}}}}}} \right)}^k}} \right\},\left\{ {1 - {{\left( {1 - {{(q)}^{1 - {\lambda _{{j_{I}}}}}}} \right)}^k}} \right\},\left\{ {{{\left( {1 - {{\left( q \right)}^{{\lambda _{{j_{F}}}}}}} \right)}^k}} \right\}
\end{array} \right\rangle  \oplus \left\langle \begin{array}{c}
\left\{ {\left[ {1 - {{\left( {1 - {{(q)}^{1 - B_{{j_{T}}}^L}}} \right)}^k},\,1 - {{\left( {1 - {{(q)}^{1 - B_{{j_{T}}}^U}}} \right)}^k}} \right]} \right\},\\
\left\{ {\left[ {1 - {{\left( {1 - {{(q)}^{1 - B_{{j_{I}}}^L}}} \right)}^k},\,1 - {{\left( {1 - {{(q)}^{1 - B_{{{j_{I}}}}^U}}} \right)}^k}} \right]} \right\},\\
\left\{ {\left[ {{{\left( {1 - {{(q)}^{B_{{j_{F}}}^L}}} \right)}^k},{{\left( {1 - {{\left( q \right)}^{B_{{j_{F}}}^U}}} \right)}^k}} \right]} \right\},\\
\left\{ {1 - {{\left( {1 - {{\left( q \right)}^{1 - {\mu _{{j_{T}}}}}}} \right)}^k}} \right\},\left\{ {1 - {{\left( {1 - {{\left( q \right)}^{1 - {\mu _{{j_{T}}}}}}} \right)}^k}} \right\},\left\{ {{{\left( {1 - {{\left( q \right)}^{{\mu _{{j_{F}}}}}}} \right)}^k}} \right\}
\end{array} \right\rangle 
$$

$$ = \left\langle \begin{array}{c}
\left\{ {\left[ {1 - {{\left( {1 - \left( {{{(q)}^{1 - A_{{j_{T}}}^L}} + {{(q)}^{1 - B_{{j_{T}}}^L}} - {{(q)}^{1 - A_{{j_{T}}}^L}}{{(q)}^{1 - B_{{j_{T}}}^L}}} \right)} \right)}^k},\,1 - {{\left( {1 - \,\left( {{{(q)}^{1 - A_{{j_{T}}}^U}} + {{(q)}^{1 - B_{{j_{T}}}^U}} - {{(q)}^{1 - A_{{j_{T}}}^U}}{{(q)}^{1 - B_{{j_{T}}}^U}}} \right)} \right)}^k}} \right]} \right\},\\
\left\{ {\left[ {1 - {{\left( {1 - \left( {{{(q)}^{1 - A_{{j_{I}}}^L}} + {{(q)}^{1 - B_{{j_{I}}}^L}} - {{(q)}^{1 - A_{{j_{I}}}^L}}{{(q)}^{1 - B_{{j_{I}}}^L}}} \right)} \right)}^k},\,1 - {{\left( {1 - \left( {{{(q)}^{1 - A_{{{j_{I}}}}^U}} + {{(q)}^{1 - B_{{{j_{I}}}}^U}} - {{(q)}^{1 - A_{{{j_{I}}}}^U}}{{(q)}^{1 - B_{{{j_{I}}}}^U}}} \right)} \right)}^k}} \right]} \right\},\\
\left\{ {\left[ {{{\left( {\left( {1 - {{(q)}^{A_{{j_{F}}}^L}}} \right)\left( {1 - {{(q)}^{B_{{j_{F}}}^L}}} \right)} \right)}^k},{{\left( {\left( {1 - {{\left( q \right)}^{A_{{j_{F}}}^U}}} \right)\left( {1 - {{\left( q \right)}^{B_{{j_{F}}}^U}}} \right)} \right)}^k}} \right]} \right\},\\
\left\{ {1 - {{\left( {1 - \left( {{{(q)}^{1 - {\lambda _{{j_{T}}}}}} + {{(q)}^{1 - {\mu _{{j_{T}}}}}} - {{(q)}^{1 - {\lambda _{{j_{T}}}}}}{{(q)}^{1 - {\mu _{{j_{T}}}}}}} \right)} \right)}^k}} \right\},\\
\left\{ {1 - {{\left( {1 - \left( {{{(q)}^{1 - {\lambda _{{j_{I}}}}}} + {{(q)}^{1 - {\mu _{{j_{I}}}}}} - {{(q)}^{1 - {\lambda _{{j_{I}}}}}}{{(q)}^{1 - {\mu _{{j_{I}}}}}}} \right)} \right)}^k}} \right\},\left\{ {{{\left( {\left( {1 - {{\left( q \right)}^{{\lambda _{{j_{F}}}}}}} \right)\left( {1 - {{\left( q \right)}^{{\mu _{{j_{F}}}}}}} \right)} \right)}^k}} \right\}
\end{array} \right\rangle .
$$

(ii)

$${\left( {{q^\alpha } \otimes {q^\beta }} \right)^k} = \left\langle \begin{array}{c}
\left\{ {\left[ {{{\left( {{{(q)}^{1 - A_{{j_{T}}}^L}}{{(q)}^{1 - B_{{j_{T}}}^L}}} \right)}^k},\,{{\left( {{{(q)}^{1 - A_{{j_{T}}}^U}}{{(q)}^{1 - B_{{j_{T}}}^U}}} \right)}^k}} \right]} \right\},\\
\left\{ {\left[ {{{\left( {{{(q)}^{1 - A_{{j_{I}}}^L}}{{(q)}^{1 - B_{{j_{I}}}^L}}} \right)}^k},\,{{\left( {{{(q)}^{1 - A_{{{j_{I}}}}^U}}{{(q)}^{1 - B_{{{j_{I}}}}^U}}} \right)}^k}} \right]} \right\},\\
\left\{ {\left[ \begin{array}{l}
1 - {\left( {1 - \left( {1 - {{(q)}^{A_{{j_{F}}}^L}} + 1 - {{(q)}^{B_{{j_{F}}}^L}} - \left( {1 - {{(q)}^{A_{{j_{F}}}^L}}} \right)\left( {1 - {{(q)}^{B_{{j_{F}}}^L}}} \right)} \right)} \right)^k},\,\\
1 - {\left( {1 - \left( {1 - {{\left( q \right)}^{A_{{j_{F}}}^U}} + 1 - {{\left( q \right)}^{B_{{j_{F}}}^U}} - \left( {1 - {{\left( q \right)}^{A_{{j_{F}}}^U}}} \right)\left( {1 - {{\left( q \right)}^{B_{{j_{F}}}^U}}} \right)} \right)} \right)^k}
\end{array} \right]} \right\},\\
\left\{ {{{\left( {{{\left( q \right)}^{1 - {\lambda _{{j_{T}}}}}}{{\left( q \right)}^{1 - {\mu _{{j_{T}}}}}}} \right)}^k}} \right\},\left\{ {{{\left( {{{\left( q \right)}^{1 - {\lambda _{{j_{I}}}}}}{{\left( q \right)}^{1 - {\mu _{{j_{I}}}}}}} \right)}^k}} \right\},\\
\left\{ {1 - {{\left( {1 - \left( {1 - {{\left( q \right)}^{{\lambda _{{j_{F}}}}}} + 1 - {{\left( q \right)}^{{\mu _{{j_{F}}}}}} - \left( {1 - {{\left( q \right)}^{{\lambda _{{j_{F}}}}}}} \right)\left( {1 - {{\left( q \right)}^{{\mu _{{j_{F}}}}}}} \right)} \right)} \right)}^k}} \right\}
\end{array} \right\rangle .
$$

$${\left( {{q^\alpha }} \right)^k} \otimes {\left( {{q^\beta }} \right)^k} = \left\langle {\scriptstyle\left\{ {\left[ {{{\left( {{{(q)}^{1 - A_{{j_{T}}}^L}}} \right)}^k},{{\left( {{{(q)}^{1 - A_{{j_{T}}}^U}}} \right)}^k}} \right]} \right\},\left\{ {\left[ {{{\left( {{{(q)}^{1 - A_{{j_{I}}}^L}}} \right)}^k},{{\left( {{{(q)}^{1 - A_{{{j_{I}}}}^U}}} \right)}^k}} \right]} \right\},\atop
{\scriptstyle\left\{ {\left[ {1 - {{\left( {1 - \left( {1 - {{(q)}^{A_{{j_{F}}}^L}}} \right)} \right)}^k},\,1 - {{\left( {1 - \left( {1 - {{\left( q \right)}^{^{A_{{j_{F}}}^U}}}} \right)} \right)}^k}} \right]} \right\},\atop
\scriptstyle\left\{ {{{\left( {{{\left( q \right)}^{^{1 - {\lambda _{{j_{T}}}}}}}} \right)}^k}} \right\},\left\{ {{{\left( {{{\left( q \right)}^{^{1 - {\lambda _{{j_{I}}}}}}}} \right)}^k}} \right\},\left\{ {1 - {{\left( {1 - \left( {1 - {{\left( q \right)}^{^{{\lambda _{{j_{F}}}}}}}} \right)} \right)}^k}} \right\}}} \right\rangle  \otimes \left\langle {\scriptstyle\left\{ {\left[ {{{\left( {{{(q)}^{1 - B_{{j_{T}}}^L}}} \right)}^k},{{\left( {{{(q)}^{1 - B_{{j_{T}}}^U}}} \right)}^k}} \right]} \right\},\left\{ {\left[ {{{\left( {{{(q)}^{1 - B_{{j_{I}}}^L}}} \right)}^k},{{\left( {{{(q)}^{1 - B_{{{j_{I}}}}^U}}} \right)}^k}} \right]} \right\},\atop
{\scriptstyle\left\{ {\left[ {1 - {{\left( {1 - \left( {1 - {{(q)}^{B_{{j_{F}}}^L}}} \right)} \right)}^k},\,1 - {{\left( {1 - \left( {1 - {{\left( q \right)}^{^{B_{{j_{F}}}^U}}}} \right)} \right)}^k}} \right]} \right\},\atop
\scriptstyle\left\{ {{{\left( {{{\left( q \right)}^{^{1 - {\mu _{{j_{T}}}}}}}} \right)}^k}} \right\},\left\{ {{{\left( {{{\left( q \right)}^{^{1 - {\mu _{{j_{I}}}}}}}} \right)}^k}} \right\},\left\{ {1 - {{\left( {1 - \left( {1 - {{\left( q \right)}^{^{{\mu _{{j_{F}}}}}}}} \right)} \right)}^k}} \right\}}} \right\rangle 
$$

$$ = \left\langle \begin{array}{c}
\left\{ {\left[ {{{\left( {{{(q)}^{1 - A_{{j_{T}}}^L}}{{(q)}^{1 - B_{{j_{T}}}^L}}} \right)}^k},\,{{\left( {{{(q)}^{1 - A_{{j_{T}}}^U}}{{(q)}^{1 - B_{{j_{T}}}^U}}} \right)}^k}} \right]} \right\},\\
\left\{ {\left[ {{{\left( {{{(q)}^{1 - A_{{j_{I}}}^L}}{{(q)}^{1 - B_{{j_{I}}}^L}}} \right)}^k},\,{{\left( {{{(q)}^{1 - A_{{{j_{I}}}}^U}}{{(q)}^{1 - B_{{{j_{I}}}}^U}}} \right)}^k}} \right]} \right\},\\
\left\{ {\left[ \begin{array}{l}
1 - {\left( {1 - \left( {1 - {{(q)}^{A_{{j_{F}}}^L}} + 1 - {{(q)}^{B_{{j_{F}}}^L}} - \left( {1 - {{(q)}^{A_{{j_{F}}}^L}}} \right)\left( {1 - {{(q)}^{B_{{j_{F}}}^L}}} \right)} \right)} \right)^k},\,\\
1 - {\left( {1 - \left( {1 - {{\left( q \right)}^{A_{{j_{F}}}^U}} + 1 - {{\left( q \right)}^{B_{{j_{F}}}^U}} - \left( {1 - {{\left( q \right)}^{A_{{j_{F}}}^U}}} \right)\left( {1 - {{\left( q \right)}^{B_{{j_{F}}}^U}}} \right)} \right)} \right)^k}
\end{array} \right]} \right\},\\
\left\{ {{{\left( {{{\left( q \right)}^{1 - {\lambda _{{j_{T}}}}}}{{\left( q \right)}^{1 - {\mu _{{j_{T}}}}}}} \right)}^k}} \right\},\left\{ {{{\left( {{{\left( q \right)}^{1 - {\lambda _{{j_{I}}}}}}{{\left( q \right)}^{1 - {\mu _{{j_{I}}}}}}} \right)}^k}} \right\},\\
\left\{ {1 - {{\left( {1 - \left( {1 - {{\left( q \right)}^{{\lambda _{{j_{F}}}}}} + 1 - {{\left( q \right)}^{{\mu _{{j_{F}}}}}} - \left( {1 - {{\left( q \right)}^{{\lambda _{{j_{F}}}}}}} \right)\left( {1 - {{\left( q \right)}^{{\mu _{{j_{F}}}}}}} \right)} \right)} \right)}^k}} \right\}
\end{array} \right\rangle 
$$

(iii)

$$\left( {{k_1} + {k_2}} \right){q^\alpha } = \left\langle \begin{array}{c}
\left\{ {\left[ {1 - {{\left( {1 - {{(q)}^{1 - A_{{j_{T}}}^L}}} \right)}^{{k_1} + {k_2}}},\,1 - {{\left( {1 - {{(q)}^{1 - A_{{j_{T}}}^U}}} \right)}^{{k_1} + {k_2}}}} \right]} \right\},\\
\left\{ {\left[ {1 - {{\left( {1 - {{(q)}^{1 - A_{{j_{I}}}^L}}} \right)}^{{k_1} + {k_2}}},\,1 - {{\left( {1 - {{(q)}^{1 - A_{{{j_{I}}}}^U}}} \right)}^{{k_1} + {k_2}}}} \right]} \right\},\\
\left\{ {\left[ {{{\left( {1 - {{(q)}^{A_{{j_{F}}}^L}}} \right)}^{{k_1} + {k_2}}},{{\left( {1 - {{\left( q \right)}^{A_{{j_{F}}}^U}}} \right)}^{{k_1} + {k_2}}}} \right]} \right\},\\
\left\{ {1 - {{\left( {1 - {{\left( q \right)}^{1 - {\lambda _{{j_{T}}}}}}} \right)}^{{k_1} + {k_2}}}} \right\},\left\{ {1 - {{\left( {1 - {{\left( q \right)}^{1 - {\lambda _{{j_{I}}}}}}} \right)}^{{k_1} + {k_2}}}} \right\},\left\{ {{{\left( {1 - {{\left( q \right)}^{{\lambda _{{j_{F}}}}}}} \right)}^{{k_1} + {k_2}}}} \right\}
\end{array} \right\rangle 
$$

$$ = \left\langle \begin{array}{c}
\left\{ {\left[ \begin{array}{l}
1 - {\left( {1 - {{(q)}^{1 - A_{{j_{T}}}^L}}} \right)^{{k_1}}} + 1 - {\left( {1 - {{(q)}^{1 - A_{{j_{T}}}^L}}} \right)^{{k_2}}} - \left( {1 - {{\left( {1 - {{(q)}^{1 - A_{{j_{T}}}^L}}} \right)}^{{k_1}}}} \right)\left( {1 - {{\left( {1 - {{(q)}^{1 - A_{{j_{T}}}^L}}} \right)}^{{k_2}}}} \right),\,\\
1 - {\left( {1 - {{(q)}^{1 - A_{{j_{T}}}^U}}} \right)^{{k_1}}} + 1 - {\left( {1 - {{(q)}^{1 - A_{{j_{T}}}^U}}} \right)^{{k_2}}} - \left( {1 - {{\left( {1 - {{(q)}^{1 - A_{{j_{T}}}^U}}} \right)}^{{k_1}}}} \right)\left( {1 - {{\left( {1 - {{(q)}^{1 - A_{{j_{T}}}^U}}} \right)}^{{k_2}}}} \right)
\end{array} \right]} \right\},\\
\left\{ {\left[ \begin{array}{l}
1 - {\left( {1 - {{(q)}^{1 - A_{{j_{I}}}^L}}} \right)^{{k_1}}} + 1 - {\left( {1 - {{(q)}^{1 - A_{{j_{I}}}^L}}} \right)^{{k_2}}} - \left( {1 - {{\left( {1 - {{(q)}^{1 - A_{{j_{I}}}^L}}} \right)}^{{k_1}}}} \right)\left( {1 - {{\left( {1 - {{(q)}^{1 - A_{{j_{I}}}^L}}} \right)}^{{k_2}}}} \right),\,\\
1 - {\left( {1 - {{(q)}^{1 - A_{{{j_{I}}}}^U}}} \right)^{{k_1}}} + 1 - {\left( {1 - {{(q)}^{1 - A_{{{j_{I}}}}^U}}} \right)^{{k_2}}} - \left( {1 - {{\left( {1 - {{(q)}^{1 - A_{{{j_{I}}}}^U}}} \right)}^{{k_1}}}} \right)\left( {1 - {{\left( {1 - {{(q)}^{1 - A_{{{j_{I}}}}^U}}} \right)}^{{k_2}}}} \right)
\end{array} \right]} \right\},\\
\left\{ {\left[ {{{\left( {1 - {{(q)}^{A_{{j_{F}}}^L}}} \right)}^{{k_1}}}{{\left( {1 - {{(q)}^{A_{{j_{F}}}^L}}} \right)}^{{k_2}}},\,{{\left( {1 - {{\left( q \right)}^{A_{{j_{F}}}^U}}} \right)}^{{k_1}}}{{\left( {1 - {{\left( q \right)}^{A_{{j_{F}}}^U}}} \right)}^{{k_2}}}} \right]} \right\},\\
\left\{ {1 - {{\left( {1 - {{\left( q \right)}^{1 - {\lambda _{{j_{T}}}}}}} \right)}^{{k_1}}} + 1 - {{\left( {1 - {{\left( q \right)}^{1 - {\lambda _{{j_{T}}}}}}} \right)}^{{k_2}}} - \left( {1 - {{\left( {1 - {{\left( q \right)}^{1 - {\lambda _{{j_{T}}}}}}} \right)}^{{k_1}}}\left( {1 - {{\left( {1 - {{\left( q \right)}^{1 - {\lambda _{{j_{T}}}}}}} \right)}^{{k_2}}}} \right)} \right)} \right\},\\
\left\{ {1 - {{\left( {1 - {{\left( q \right)}^{1 - {\lambda _{{j_{I}}}}}}} \right)}^{{k_1}}} + 1 - {{\left( {1 - {{\left( q \right)}^{1 - {\lambda _{{j_{I}}}}}}} \right)}^{{k_2}}} - \left( {1 - {{\left( {1 - {{\left( q \right)}^{1 - {\lambda _{{j_{I}}}}}}} \right)}^{{k_1}}}\left( {1 - {{\left( {1 - {{\left( q \right)}^{1 - {\lambda _{{j_{I}}}}}}} \right)}^{{k_2}}}} \right)} \right)} \right\},\\
\left\{ {{{\left( {1 - {{\left( q \right)}^{{\lambda _{{j_{F}}}}}}} \right)}^{{k_1}}}{{\left( {1 - {{\left( q \right)}^{{\lambda _{{j_{F}}}}}}} \right)}^{{k_2}}}} \right\}
\end{array} \right\rangle ,
$$

$$ = \left\langle \begin{array}{c}
\left\{ {\left[ {1 - {{\left( {1 - {{(q)}^{1 - A_{{j_{T}}}^L}}} \right)}^{{k_1} + {k_2}}},\,1 - {{\left( {1 - {{(q)}^{1 - A_{{j_{T}}}^U}}} \right)}^{{k_1} + {k_2}}}} \right]} \right\},\\
\left\{ {\left[ {1 - {{\left( {1 - {{(q)}^{1 - A_{{j_{I}}}^L}}} \right)}^{{k_1} + {k_2}}},\,1 - {{\left( {1 - {{(q)}^{1 - A_{{{j_{I}}}}^U}}} \right)}^{{k_1} + {k_2}}}} \right]} \right\},\\
\left\{ {\left[ {{{\left( {1 - {{(q)}^{A_{{j_{F}}}^L}}} \right)}^{{k_1} + {k_2}}},{{\left( {1 - {{\left( q \right)}^{A_{{j_{F}}}^U}}} \right)}^{{k_1} + {k_2}}}} \right]} \right\},\\
\left\{ {1 - {{\left( {1 - {{\left( q \right)}^{1 - {\lambda _{{j_{T}}}}}}} \right)}^{{k_1} + {k_2}}}} \right\},\left\{ {1 - {{\left( {1 - {{\left( q \right)}^{1 - {\lambda _{{j_{I}}}}}}} \right)}^{{k_1} + {k_2}}}} \right\},\left\{ {{{\left( {1 - {{\left( q \right)}^{{\lambda _{{j_{F}}}}}}} \right)}^{{k_1} + {k_2}}}} \right\}
\end{array} \right\rangle 
$$

(iv)

$$ \begin{aligned} \left( {q^{\alpha } } \right)^{{k_{1} }} \otimes \left( {q^{\alpha } } \right)^{{k_{2} }} &= \left\langle \substack{ \left\{ {\left[ {\left( {(q)^{{1 - A_{{j_{{{T} }} }}^{L} }} } \right)^{{k_{1} }} ,\left( {(q)^{{1 - A_{{j_{{{T} }} }}^{U} }} } \right)^{{k_{1} }} } \right]} \right\},\left\{ {\left[ {\left( {(q)^{{1 - A_{{j_{{{I} }} }}^{L} }} } \right)^{{k_{1} }} ,\left( {(q)^{{1 - A_{{{{j_{{{I} }} }} }}^{U} }} } \right)^{{k_{1} }} } \right]} \right\}, \\ \left\{ {\left[ {1 - \left( {1 - \left( {1 - (q)^{{A_{{j_{{{F} }} }}^{L} }} } \right)} \right)^{{k_{1} }} ,\,1 - \left( {1 - \left( {1 - \left( q \right)^{{^{{A_{{j_{{{F} }} }}^{U} }} }} } \right)} \right)^{{k_{1} }} } \right]} \right\}, \\ \left\{ {\left( {\left( q \right)^{{^{{1 - \lambda_{{j_{{{T} }} }} }} }} } \right)^{{k_{1} }} } \right\},\left\{ {\left( {\left( q \right)^{{^{{1 - \lambda_{{j_{{{I} }} }} }} }} } \right)^{{k_{1} }} } \right\},\left\{ {1 - \left( {1 - \left( {1 - \left( q \right)^{{^{{\lambda_{{j_{{{F} }} }} }} }} } \right)} \right)^{{k_{1} }} } \right\} } \right\rangle \otimes \left\langle \substack{ \left\{ {\left[ {\left( {(q)^{{1 - A_{{j_{{{T} }} }}^{L} }} } \right)^{{k_{2} }} ,\left( {(q)^{{1 - A_{{j_{{{T} }} }}^{U} }} } \right)^{{k_{2} }} } \right]} \right\},\left\{ {\left[ {\left( {(q)^{{1 - A_{{j_{{{I} }} }}^{L} }} } \right)^{{k_{2} }} ,\left( {(q)^{{1 - A_{{{{j_{{{I} }} }} }}^{U} }} } \right)^{{k_{2} }} } \right]} \right\}, \\ \left\{ {\left[ {1 - \left( {1 - \left( {1 - (q)^{{A_{{j_{{{F} }} }}^{L} }} } \right)} \right)^{{k_{2} }} ,\,1 - \left( {1 - \left( {1 - \left( q \right)^{{^{{A_{{j_{{{F} }} }}^{U} }} }} } \right)} \right)^{{k_{2} }} } \right]} \right\}, \\ \left\{ {\left( {\left( q \right)^{{^{{1 - \lambda_{{j_{{{T} }} }} }} }} } \right)^{{k_{2} }} } \right\},\left\{ {\left( {\left( q \right)^{{^{{1 - \lambda_{{j_{{{I} }} }} }} }} } \right)^{{k_{2} }} } \right\},\left\{ {1 - \left( {1 - \left( {1 - \left( q \right)^{{^{{\lambda_{{j_{{{F} }} }} }} }} } \right)} \right)^{{k_{2} }} } \right\} } \right\rangle \\ &= \left\langle \begin{gathered} \left\{ {\left[ {\left( {(q)^{{1 - A_{{j_{{{T} }} }}^{L} }} } \right)^{{k_{1} + k_{2} }} ,\left( {(q)^{{1 - A_{{j_{{{T} }} }}^{U} }} } \right)^{{k_{1} + k_{2} }} } \right]} \right\},\left\{ {\left[ {\left( {(q)^{{1 - A_{{j_{{{I} }} }}^{L} }} } \right)^{{k_{1} + k_{2} }} ,\left( {(q)^{{1 - A_{{{{j_{{{I} }} }} }}^{U} }} } \right)^{{k_{1} + k_{2} }} } \right]} \right\}, \\ \left\{ {\left[ {1 - \left( {1 - \left( {1 - (q)^{{A_{{j_{{{F} }} }}^{L} }} } \right)} \right)^{{k_{1} + k_{2} }} ,\,1 - \left( {1 - \left( {1 - \left( q \right)^{{^{{A_{{j_{{{F} }} }}^{U} }} }} } \right)} \right)^{{k_{1} + k_{2} }} } \right]} \right\}, \\ \left( {\left( q \right)^{{^{{1 - \lambda_{{j_{{{T} }} }} }} }} } \right)^{{k_{1} + k_{2} }} ,\left\{ {\left( {\left( q \right)^{{^{{1 - \lambda_{{j_{{{I} }} }} }} }} } \right)^{{k_{1} + k_{2} }} } \right\},\left\{ {1 - \left( {1 - \left( {1 - \left( q \right)^{{^{{\lambda_{{j_{{{F} }} }} }} }} } \right)} \right)^{{k_{1} + k_{2} }} } \right\} \\ \end{gathered} \right\rangle = \left( {q^{\alpha } } \right)^{{k_{1} + k_{2} }} \end{aligned} $$

(v)

$$ \begin{gathered} q^{\alpha } \otimes q_{1}^{\alpha } = \left\langle \begin{gathered} \left\{ {\left[ {(q)^{{1 - A_{{j_{{{T} }} }}^{L} }} ,(q)^{{1 - A_{{j_{{{T} }} }}^{U} }} } \right]} \right\},\left\{ {\left[ {(q)^{{1 - A_{{j_{{{I} }} }}^{L} }} ,(q)^{{1 - A_{{{{j_{{{I} }} }} }}^{U} }} } \right]} \right\}, \\ \left\{ {\left[ {1 - (q)^{{A_{{j_{{{F} }} }}^{L} }} ,\,1 - \left( q \right)^{{A_{{j_{{{F} }} }}^{U} }} } \right]} \right\}, \\ \left\{ {\left( q \right)^{{1 - \lambda_{{j_{{{T} }} }} }} } \right\},\left\{ {\left( q \right)^{{1 - \lambda_{{j_{{{I} }} }} }} } \right\},\left\{ {1 - \left( q \right)^{{\lambda_{{j_{{{F} }} }} }} } \right\} \\ \end{gathered} \right\rangle \otimes \left\langle \begin{gathered} \left\{ {\left[ {(q_{1} )^{{1 - A_{{j_{{{T} }} }}^{L} }} ,(q_{1} )^{{1 - A_{{j_{{{T} }} }}^{U} }} } \right]} \right\},\left\{ {\left[ {(q_{1} )^{{1 - A_{{j_{{{I} }} }}^{L} }} ,(q_{1} )^{{1 - A_{{{{j_{{{I} }} }} }}^{U} }} } \right]} \right\}, \\ \left\{ {\left[ {1 - (q_{1} )^{{A_{{j_{{{F} }} }}^{L} }} ,\,1 - \left( {q_{1} } \right)^{{A_{{j_{{{F} }} }}^{U} }} } \right]} \right\}, \\ \left\{ {\left( {q_{1} } \right)^{{1 - \lambda_{{j_{{{T} }} }} }} } \right\},\left\{ {\left( {q_{1} } \right)^{{1 - \lambda_{{j_{{{I} }} }} }} } \right\},\left\{ {1 - \left( {q_{1} } \right)^{{\lambda_{{j_{{{F} }} }} }} } \right\} \\ \end{gathered} \right\rangle \hfill \\ = \left\langle \begin{gathered} \left\{ {\left[ {(q)^{{1 - A_{{j_{{{T} }} }}^{L} }} (q_{1} )^{{1 - A_{{j_{{{T} }} }}^{L} }} ,\,(q)^{{1 - A_{{j_{{{T} }} }}^{U} }} (q_{1} )^{{1 - A_{{j_{{{T} }} }}^{U} }} } \right]} \right\},\left\{ {\left[ {(q)^{{1 - A_{{j_{{{I} }} }}^{L} }} (q_{1} )^{{1 - A_{{j_{{{I} }} }}^{L} }} ,\,(q)^{{1 - A_{{{{j_{{{I} }} }} }}^{U} }} (q_{1} )^{{1 - A_{{{{j_{{{I} }} }} }}^{U} }} } \right]} \right\}, \\ \left\{ {\left[ \begin{gathered} 1 - (q)^{{A_{{j_{{{F} }} }}^{L} }} + 1 - (q_{1} )^{{A_{{j_{{{F} }} }}^{L} }} - \left( {1 - (q)^{{A_{{j_{{{F} }} }}^{L} }} } \right)\left( {1 - (q_{1} )^{{A_{{j_{{{F} }} }}^{L} }} } \right),\, \hfill \\ 1 - \left( q \right)^{{A_{{j_{{{F} }} }}^{U} }} + \,1 - \left( {q_{1} } \right)^{{A_{{j_{{{F} }} }}^{U} }} - \left( {1 - \left( q \right)^{{A_{{j_{{{F} }} }}^{U} }} } \right)\left( {\,1 - \left( {q_{1} } \right)^{{A_{{j_{{{F} }} }}^{U} }} } \right) \hfill \\ \end{gathered} \right]} \right\}, \\ \left\{ {\left( q \right)^{{1 - \lambda_{{j_{{{T} }} }} }} \left( {q_{1} } \right)^{{1 - \lambda_{{j_{{{T} }} }} }} } \right\},\left\{ {\left( q \right)^{{\lambda_{{j_{{{I} }} }} }} \left( {q_{1} } \right)^{{\lambda_{{j_{{{I} }} }} }} } \right\}, \\ \left\{ {1 - \left( q \right)^{{\lambda_{{j_{{{F} }} }} }} + 1 - \left( {q_{1} } \right)^{{\lambda_{{j_{{{F} }} }} }} - \left( {1 - \left( q \right)^{{\lambda_{{j_{{{F} }} }} }} } \right)\left( {1 - \left( {q_{1} } \right)^{{\lambda_{{j_{{{F} }} }} }} } \right)} \right\} \\ \end{gathered} \right\rangle . \hfill \\ = \left\langle \begin{gathered} \left\{ {\left[ {(qq_{1} )^{{1 - A_{{j_{{{T} }} }}^{L} }} ,(q)^{{1 - A_{{j_{{{T} }} }}^{U} }} } \right]} \right\},\left\{ {\left[ {(qq_{1} )^{{1 - A_{{j_{{{I} }} }}^{L} }} ,(qq_{1} )^{{1 - A_{{{{j_{{{I} }} }} }}^{U} }} } \right]} \right\} \\ ,\left\{ {\left[ {1 - (qq_{1} )^{{A_{{j_{{{F} }} }}^{L} }} ,\,1 - \left( {qq_{1} } \right)^{{A_{{j_{{{F} }} }}^{U} }} } \right]} \right\}, \\ \left\{ {\left( {qq_{1} } \right)^{{1 - \lambda_{{j_{{{T} }} }} }} } \right\},\left\{ {\left( {qq_{1} } \right)^{{1 - \lambda_{{j_{{{I} }} }} }} } \right\},\left\{ {1 - \left( {qq_{1} } \right)^{{\lambda_{{j_{{{F} }} }} }} } \right\} \\ \end{gathered} \right\rangle \hfill \\ = \left( {qq_{1} } \right)^{\alpha } \hfill \\ \end{gathered} $$

## Exponential aggregation operators

### Definition 16

Let $\left\{ {\alpha_{(k)} = \left\langle {A_{(k)} ,\,\lambda_{(k)} } \right\rangle } \right\}$ be a collection of NCHFNs and $q_{k} \in (0,1)$ be real numbers, then Neutrosophic cubic hesitant fuzzy weighted exponential aggregation operator is defined as $NCHWEA(\alpha_{1} ,\,\alpha_{2} ,...,\,\alpha_{n} ) = \mathop \otimes \limits_{j = 1}^{n} \left( {q_{j} } \right)^{{\alpha_{j} }}$ and $\left\{ {\alpha_{(k)} = \left\langle {A_{(k)} ,\,\lambda_{(k)} } \right\rangle } \right\}$ are the exponential weighting vector of attribute values $q_{k} \in (0,1)$.

### Theorem 6

*Let* $\left\{ {\alpha_{(k)} = \left\langle {A_{(k)} ,\,\lambda_{(k)} } \right\rangle } \right\}$* be a collection of NCHFNs and* $q_{k} \in (0,1)$* be real numbers, then Neutrosophic cubic hesitant fuzzy weighted exponential aggregation operator is*

$$NCHFWEA({\alpha _1},\,{\alpha _2}, \ldots ,\,{\alpha _m}) = \left\langle {\begin{array}{*{20}{c}}
{\left\{ {\left[ {\prod\limits_{k = 1}^m {{{\left( {{q_k}} \right)}^{1 - A_{{j_{{T_{(k)}}}}}^L}},} \prod\limits_{k = 1}^m {{{\left( {{q_k}} \right)}^{1 - A_{{j_{{T_{(k)}}}}}^U}}} } \right]} \right\},\left\{ {\left[ {\prod\limits_{k = 1}^m {{{\left( {{q_k}} \right)}^{1 - A_{{j_{{I_{(k)}}}}}^L}}} ,\prod\limits_{k = 1}^m {{{\left( {{q_k}} \right)}^{1 - A_{{j_{{I_{(k)}}}}}^U}}} } \right]} \right\},}\\
{\left\{ {\left[ {1 - \prod\limits_{k = 1}^m {{{\left( {{q_k}} \right)}^{A_{{j_{{F_{(k)}}}}}^L}}} ,1 - \prod\limits_{k = 1}^m {{{\left( {{q_k}} \right)}^{A_{{j_{{F_{(k)}}}}}^U}}} } \right]} \right\},\left\{ {\prod\limits_{k = 1}^m {{{\left( {{q_k}} \right)}^{1 - {\lambda _{{j_{{T_{(k)}}}}}}}}} } \right\}}\\
{\left\{ {\prod\limits_{k = 1}^m {{{\left( {{q_k}} \right)}^{1 - {\lambda _{{j_{{I_{(k)}}}}}}}}} } \right\},\left\{ {1 - \prod\limits_{k = 1}^m {{{\left( {{q_k}} \right)}^{{\lambda _{{j_{{F_{(k)}}}}}}}}} } \right\}}
\end{array}} \right\rangle\,\, {\text{and}}  
$$

$\left\{ {\alpha_{(k)} = \left\langle {A_{(k)} ,\,\lambda_{(k)} } \right\rangle } \right\}$* are the exponential weighting vectors of attribute values* $q_{k} \in (0,1)$*. Furthermore* $NCHFHG(\alpha_{1} ,\,\alpha_{2} , \ldots ,\,\alpha_{m} )$ *is also a neutrosophic cubic hesitant fuzzy value.*

### Proof

Using induction we have,$NCHFWEA(\alpha_{1} ,\,\alpha_{2} ) = \mathop \otimes \limits_{j = 1}^{2} \left( {q_{j} } \right)^{{\alpha_{j} }}$.

$$\begin{array}{l}
 = \left\langle \begin{array}{c}
\left\{ {\left[ {{{({q_1})}^{1 - A_{{j_{{{T_{(1)}}}}}}^L}},{{({q_1})}^{1 - A_{{j_{{{T_{(1)}}}}}}^U}}} \right]} \right\},\left\{ {\left[ {{{({q_1})}^{1 - A_{{j_{{{I_{(1)}}}}}}^L}},{{({q_1})}^{1 - A_{{{j_{{{I_{(1)}}}}}}}^U}}} \right]} \right\},\\
\left\{ {\left[ {1 - {{({q_1})}^{A_{{j_{{{F_{(1)}}}}}}^L}},\,1 - {{\left( {{q_1}} \right)}^{A_{{j_{{{F_{(1)}}}}}}^U}}} \right]} \right\},\\
\left\{ {{{\left( {{q_1}} \right)}^{1 - {\lambda _{{j_{{{T_{(1)}}}}}}}}}} \right\},\left\{ {{{\left( {{q_1}} \right)}^{1 - {\lambda _{{j_{{{I_{(1)}}}}}}}}}} \right\},\left\{ {1 - {{\left( {{q_1}} \right)}^{{\lambda _{{j_{{{F_{(1)}}}}}}}}}} \right\}
\end{array} \right\rangle  \otimes \left\langle \begin{array}{c}
\left\{ {\left[ {{{({q_2})}^{1 - A_{{j_{{{T_{(2)}}}}}}^L}},{{({q_2})}^{1 - A_{{j_{{{T_{(2)}}}}}}^U}}} \right]} \right\},\left\{ {\left[ {{{({q_2})}^{1 - A_{{j_{{{I_{(2)}}}}}}^L}},{{({q_2})}^{1 - A_{{{j_{{{I_{(2)}}}}}}}^U}}} \right]} \right\},\\
\left\{ {\left[ {1 - {{({q_2})}^{A_{{j_{{{F_{(2)}}}}}}^L}},\,1 - {{\left( {{q_2}} \right)}^{A_{{j_{{{F_{(2)}}}}}}^U}}} \right]} \right\},\\
\left\{ {{{\left( {{q_2}} \right)}^{1 - {\lambda _{{j_{{{T_{(2)}}}}}}}}}} \right\},\left\{ {{{\left( {{q_2}} \right)}^{1 - {\lambda _{{j_{{{I_{(2)}}}}}}}}}} \right\},\left\{ {1 - {{\left( {{q_2}} \right)}^{{\lambda _{{j_{{{F_{(2)}}}}}}}}}} \right\}
\end{array} \right\rangle \\
 = \left\langle \begin{array}{c}
\left\{ {\left[ {{{({q_1})}^{1 - A_{{j_{{{T_{(1)}}}}}}^L}}{{({q_2})}^{1 - A_{{j_{{{T_{(2)}}}}}}^L}},{{({q_1})}^{1 - A_{{j_{{{T_{(1)}}}}}}^U}}{{({q_2})}^{1 - A_{{j_{{{T_{(2)}}}}}}^U}}} \right]} \right\},\left\{ {\left[ {{{({q_1})}^{1 - A_{{j_{{{I_{(1)}}}}}}^L}}{{({q_2})}^{1 - A_{{j_{{{I_{(2)}}}}}}^L}},{{({q_1})}^{1 - A_{{{j_{{{I_{(1)}}}}}}}^U}}{{({q_2})}^{1 - A_{{{j_{{{I_{(2)}}}}}}}^U}}} \right]} \right\},\\
\left\{ {\left[ \begin{array}{l}
1 - {({q_1})^{A_{{j_{{{F_{(1)}}}}}}^L}} + 1 - {({q_2})^{A_{{j_{{{F_{(2)}}}}}}^L}} - \left( {1 - {{({q_1})}^{A_{{j_{{{F_{(1)}}}}}}^L}}} \right)\left( {1 - {{({q_2})}^{A_{{j_{{{F_{(2)}}}}}}^L}}} \right),\,\\
1 - {\left( {{q_1}} \right)^{A_{{j_{{{F_{(1)}}}}}}^U}} + 1 - {\left( {{q_2}} \right)^{A_{{j_{{{F_{(2)}}}}}}^U}} - \left( {1 - {{\left( {{q_1}} \right)}^{A_{{j_{{{F_{(1)}}}}}}^U}}} \right)\left( {1 - {{\left( {{q_2}} \right)}^{A_{{j_{{{F_{(2)}}}}}}^U}}} \right)
\end{array} \right]} \right\},\\
\left\{ {{{\left( {{q_1}} \right)}^{1 - {\lambda _{{j_{{{T_{(1)}}}}}}}}}{{\left( {{q_2}} \right)}^{1 - {\lambda _{{j_{{{T_{(2)}}}}}}}}}} \right\},\\
\left\{ {{{\left( {{q_1}} \right)}^{1 - {\lambda _{{j_{{{I_{(1)}}}}}}}}}{{\left( {{q_2}} \right)}^{1 - {\lambda _{{j_{{{I_{(2)}}}}}}}}}} \right\},\left\{ {1 - {{\left( {{q_1}} \right)}^{{\lambda _{{j_{{{I_{(1)}}}}}}}}} + 1 - {{\left( {{q_2}} \right)}^{{\lambda _{{j_{{{I_{(2)}}}}}}}}} - \left( {1 - {{\left( {{q_1}} \right)}^{{\lambda _{{j_{{{F_{(1)}}}}}}}}}} \right)\left( {1 - {{\left( {{q_2}} \right)}^{{\lambda _{{j_{{{F_{(2)}}}}}}}}}} \right)} \right\}
\end{array} \right\rangle \\
 = \left\langle {\begin{array}{*{20}{c}}
{\left\{ {\left[ {\prod\limits_{k = 1}^2 {{{\left( {{q_k}} \right)}^{1 - A_{{j_{{T_{(k)}}}}}^L}}} ,\prod\limits_{k = 1}^2 {{{\left( {{q_k}} \right)}^{1 - A_{{j_{{T_{(k)}}}}}^U}}} } \right]} \right\},\left\{ {\left[ {\prod\limits_{k = 1}^2 {{{\left( {{q_k}} \right)}^{1 - A_{{j_{{I_{(k)}}}}}^L}}} ,\prod\limits_{k = 1}^2 {{{\left( {{q_k}} \right)}^{1 - A_{{j_{{I_{(k)}}}}}^U}}} } \right]} \right\},}\\
{\left\{ {\left[ {1 - \prod\limits_{k = 1}^2 {{{\left( {{q_k}} \right)}^{A_{{j_{{F_{(k)}}}}}^L}}} ,1 - \prod\limits_{k = 1}^2 {{{\left( {{q_k}} \right)}^{A_{{j_{{F_{(k)}}}}}^U}}} } \right]} \right\},\left\{ {\prod\limits_{k = 1}^2 {{{\left( {{q_k}} \right)}^{1 - {\lambda _{{j_{{T_{(k)}}}}}}}}} } \right\},}\\
{\left\{ {\prod\limits_{k = 1}^2 {{{\left( {{q_k}} \right)}^{1 - {\lambda _{{j_{{I_{(k)}}}}}}}}} } \right\},\left\{ {1 - \prod\limits_{k = 1}^2 {{{\left( {{q_k}} \right)}^{{\lambda _{{j_{{F_{(k)}}}}}}}}} } \right\}}
\end{array}} \right\rangle 
\end{array}
$$

For m = n we have

$$NCHFWEA({\alpha _1},\,{\alpha _2}, \ldots ,\,{\alpha _n}) = \left\langle {\begin{array}{*{20}{c}}
{\left\{ {\left[ {\prod\limits_{k = 1}^n {{{\left( {{q_k}} \right)}^{1 - A_{{j_{{T_{(k)}}}}}^L}}} ,\prod\limits_{k = 1}^n {{{\left( {{q_k}} \right)}^{1 - A_{{j_{{T_{(k)}}}}}^U}}} } \right]} \right\},\left\{ {\left[ {\prod\limits_{k = 1}^n {{{\left( {{q_k}} \right)}^{1 - A_{{j_{{I_{(k)}}}}}^L}}} ,\prod\limits_{k = 1}^n {{{\left( {{q_k}} \right)}^{1 - A_{{j_{{I_{(k)}}}}}^U}}} } \right]} \right\}}\\
{\left\{ {\left[ {1 - \prod\limits_{k = 1}^n {{{\left( {{q_k}} \right)}^{A_{{j_{{F_{(k)}}}}}^L}}} ,1 - \prod\limits_{k = 1}^n {{{\left( {{q_k}} \right)}^{A_{{j_{{F_{(k)}}}}}^U}}} } \right]} \right\},\left\{ {\prod\limits_{k = 1}^n {{{\left( {{q_k}} \right)}^{1 - {\lambda _{{j_{{T_{(k)}}}}}}}}} } \right\}}\\
{\left\{ {\prod\limits_{k = 1}^n {{{\left( {{q_k}} \right)}^{1 - {\lambda _{{j_{{I_{(k)}}}}}}}}} } \right\},\left\{ {1 - \prod\limits_{k = 1}^n {{{\left( {{q_k}} \right)}^{{\lambda _{{j_{{F_{(k)}}}}}}}}} } \right\}}
\end{array}} \right\rangle 
$$

we prove for m = n + 1

$$\begin{array}{l}
NCHFWEA({\alpha _1},\,{\alpha _2}, \ldots ,\,{\alpha _{n + 1}}) = \left\langle {\begin{array}{*{20}{l}}
{\left\{ {\left[ {\prod\limits_{k = 1}^n {{{\left( {{q_k}} \right)}^{1 - A_{{j_{{T_{(k)}}}}}^L}}} ,\prod\limits_{k = 1}^n {{{\left( {{q_k}} \right)}^{1 - A_{{j_{{T_{(k)}}}}}^U}}} } \right]} \right\},}\\
{\left\{ {\left[ {\prod\limits_{k = 1}^n {{{\left( {{q_k}} \right)}^{1 - A_{{j_{{I_{(k)}}}}}^L}}} ,\prod\limits_{k = 1}^n {{{\left( {{q_k}} \right)}^{1 - A_{{j_{{I_{(k)}}}}}^U}}} } \right]} \right\},}\\
{\left\{ {\left[ {1 - \prod\limits_{k = 1}^n {{{\left( {{q_k}} \right)}^{A_{{j_{{F_{(k)}}}}}^L}}} ,1 - \prod\limits_{k = 1}^n {{{\left( {{q_k}} \right)}^{A_{{j_{{F_{(k)}}}}}^U}}} } \right]} \right\},}\\
{\left\{ {\prod\limits_{k = 1}^n {{{\left( {{q_k}} \right)}^{1 - {\lambda _{{j_{{T_{(k)}}}}}}}}} } \right\},}\\
{\left\{ {\prod\limits_{k = 1}^n {{{\left( {{q_k}} \right)}^{1 - {\lambda _{{j_{{I_{(k)}}}}}}}}} } \right\},\left\{ {1 - \prod\limits_{k = 1}^n {{{\left( {{q_k}} \right)}^{{\lambda _{{j_{{F_{(k)}}}}}}}}} } \right\},}
\end{array}} \right\rangle  \otimes \left\langle \begin{array}{c}
\left\{ {\left[ {{{({q_{n + 1}})}^{1 - A_{{j_{{{T_{(n + 1)}}}}}}^L}},{{({q_{n + 1}})}^{1 - A_{{j_{{{T_{(n + 1)}}}}}}^U}}} \right]} \right\},\\
\left\{ {\left[ {{{({q_{n + 1}})}^{1 - A_{{j_{{{I_{(n + 1)}}}}}}^L}},{{({q_{n + 1}})}^{1 - A_{{{j_{{{I_{(n + 1)}}}}}}}^U}}} \right]} \right\},\\
\left\{ {\left[ {1 - {{({q_{n + 1}})}^{A_{{j_{{{F_{(n + 1)}}}}}}^L}},\,1 - {{\left( {{q_{n + 1}}} \right)}^{A_{{j_{{{F_{(n + 1)}}}}}}^U}}} \right]} \right\},\\
\left\{ {{{\left( {{q_{n + 1}}} \right)}^{1 - {\lambda _{{j_{{{T_{(n + 1)}}}}}}}}}} \right\},\\
\left\{ {{{\left( {{q_{n + 1}}} \right)}^{1 - {\lambda _{{j_{{{I_{(n + 1)}}}}}}}}}} \right\},\left\{ {1 - {{\left( {{q_{n + 1}}} \right)}^{{\lambda _{{j_{{{F_{(n + 1)}}}}}}}}}} \right\}
\end{array} \right\rangle \\
 = \left\langle {\begin{array}{*{20}{l}}
{\left\{ {\left[ {\prod\limits_{k = 1}^n {{{\left( {{q_k}} \right)}^{1 - A_{{j_{{T_{(k)}}}}}^L}}{{({q_{n + 1}})}^{1 - A_{{j_{{{T_{(n + 1)}}}}}}^L}}} ,\prod\limits_{k = 1}^n {{{\left( {{q_k}} \right)}^{1 - A_{{j_{{T_{(k)}}}}}^U}}{{({q_{n + 1}})}^{1 - A_{{j_{{{T_{(n + 1)}}}}}}^U}}} } \right]} \right\}}\\
{\left\{ {\left[ {\prod\limits_{k = 1}^n {{{\left( {{q_k}} \right)}^{1 - A_{{j_{{I_{(k)}}}}}^L}}{{({q_{n + 1}})}^{1 - A_{{j_{{{I_{(n + 1)}}}}}}^L}}} ,\prod\limits_{k = 1}^n {{{\left( {{q_k}} \right)}^{1 - A_{{j_{{I_{(k)}}}}}^U}}{{({q_{n + 1}})}^{1 - A_{{{j_{{{I_{(n + 1)}}}}}}}^U}}} } \right]} \right\}}\\
{\left\{ {\left[ {\begin{array}{*{20}{c}}
{1 - \prod\limits_{k = 1}^n {{{\left( {{q_k}} \right)}^{A_{{j_{{F_{(k)}}}}}^L}} + 1 - {{({q_{n + 1}})}^{A_{{j_{{{F_{(n + 1)}}}}}}^L}}}  - \left( {1 - \prod\limits_{k = 1}^n {{{\left( {{q_k}} \right)}^{A_{{j_{{F_{(k)}}}}}^L}}} } \right)\left( {1 - {{({q_{n + 1}})}^{A_{{j_{{{F_{(n + 1)}}}}}}^L}}} \right)}\\
{1 - \prod\limits_{k = 1}^n {{{\left( {{q_k}} \right)}^{A_{{j_{{F_{(k)}}}}}^U}} + 1 - {{\left( {{q_{n + 1}}} \right)}^{A_{{j_{{{F_{(n + 1)}}}}}}^U}}}  - \left( {1 - \prod\limits_{k = 1}^n {{{\left( {{q_k}} \right)}^{A_{{j_{{F_{(k)}}}}}^U}}} } \right)\left( {1 - {{\left( {{q_{n + 1}}} \right)}^{A_{{j_{{{F_{(n + 1)}}}}}}^U}}} \right)}
\end{array}} \right]} \right\}}\\
{\left\{ {\prod\limits_{k = 1}^n {{{\left( {{q_k}} \right)}^{1 - {\lambda _{{j_{{T_{(k)}}}}}}}}{{\left( {{q_{n + 1}}} \right)}^{1 - {\lambda _{{j_{{{T_{(n + 1)}}}}}}}}}} } \right\},\left\{ {\prod\limits_{k = 1}^n {{{\left( {{q_k}} \right)}^{1 - {\lambda _{{j_{{I_{(k)}}}}}}}}{{\left( {{q_{n + 1}}} \right)}^{1 - {\lambda _{{j_{{{I_{(n + 1)}}}}}}}}}} } \right\}}\\
{\left\{ {1 - \prod\limits_{k = 1}^n {{{\left( {{q_k}} \right)}^{{\lambda _{{j_{{F_{(k)}}}}}}}} + 1 - {{\left( {{q_{n + 1}}} \right)}^{{\lambda _{{j_{{{F_{(n + 1)}}}}}}}}}}  - \left( {1 - \prod\limits_{k = 1}^n {{{\left( {{q_k}} \right)}^{{\lambda _{{j_{{F_{(k)}}}}}}}}} } \right)\left( {1 - {{\left( {{q_{n + 1}}} \right)}^{{\lambda _{{j_{{{F_{(n + 1)}}}}}}}}}} \right)} \right\}}
\end{array}} \right\rangle \\
 = \left\langle {\begin{array}{*{20}{l}}
{\left\{ {\left[ {\prod\limits_{k = 1}^{n + 1} {{{\left( {{q_k}} \right)}^{1 - A_{{j_{{T_{(k)}}}}}^L}}} ,\prod\limits_{k = 1}^{n + 1} {{{\left( {{q_k}} \right)}^{1 - A_{{j_{{T_{(k)}}}}}^U}}} } \right]} \right\},}\\
{\left\{ {\left[ {\prod\limits_{k = 1}^{n + 1} {{{\left( {{q_k}} \right)}^{1 - A_{{j_{{I_{(k)}}}}}^L}}} ,\prod\limits_{k = 1}^{n + 1} {{{\left( {{q_k}} \right)}^{1 - A_{{j_{{I_{(k)}}}}}^U}}} } \right]} \right\},}\\
{\left\{ {\left[ {1 - \prod\limits_{k = 1}^{n + 1} {{{\left( {{q_k}} \right)}^{A_{{j_{{F_{(k)}}}}}^L}}} ,1 - \prod\limits_{k = 1}^{n + 1} {{{\left( {{q_k}} \right)}^{A_{{j_{{F_{(k)}}}}}^U}}} } \right]} \right\},}\\
{\left\{ {\prod\limits_{k = 1}^{n + 1} {{{\left( {{q_k}} \right)}^{1 - {\lambda _{{j_{{T_{(k)}}}}}}}}} } \right\},}\\
{\left\{ {\prod\limits_{k = 1}^{n + 1} {{{\left( {{q_k}} \right)}^{1 - {\lambda _{{j_{{I_{(k)}}}}}}}}} } \right\},\left\{ {1 - \prod\limits_{k = 1}^{n + 1} {{{\left( {{q_k}} \right)}^{{\lambda _{{j_{{F_{(k)}}}}}}}}} } \right\}}
\end{array}} \right\rangle 
\end{array}
$$

## Applications of neutrosophic cubic hesitant fuzzy weighted exponential aggregation operator to MADM and ME-MADM problems

Many methods in MADM ignore the uncertainty and hence yield the results which are unreliable. In this section we construct algorithms using the exponential aggregation (NCHFWE) for MADM and ME-MADM problems.

**Algorithm 6.1 (MADM problems)**

**Step 1: Identification of alternatives and attributes.**

Let $\left\{{F}_{1},{F}_{2},\dots ,{F}_{r}\right\}$ be the set of *r* alternatives, $\left\{{K}_{1},{K}_{2},\dots ,{K}_{s}\right\}$ be *s* attributes. The NCHFS ${\alpha }_{j}$ is used as weight for the attribute ${K}_{j}$. A decision matrix is $D=({d}_{ij})$ consisting fuzzy values, where ${d}_{ij}$ represent the preference of alternative ${F}_{i}$ corresponding to attribute ${K}_{j}$.

**Step 2. Allocation of weights to attributes**

The NCHF value ${{\alpha }_{j}}^{(k)}$ is used as weight assigned to attribute ${K}_{j}$ by expert ${E}_{k}$.

**Step 3. Computation of weighted aggregated values**

Using NCHFWEA operators, we compute the aggregated values ${d}_{j}{^{\prime}}s$ (j = 1,…,r) of alternatives ${F}_{j}{^{\prime}}s$.

**Step 4. Ranking of Alternatives**

We calculate the scores ${S(d}_{j});j=1,\dots ,r$ of the alternatives ${F}_{j};i=1,\dots ,r$. Using scores ${S(d}_{i});i=1,\dots ,n,$ we rank the alternatives ${F}_{i};i=1,\dots ,n$. If scores of two alternatives are equal, then we use accuracy function for ranking and if they have same accuracy, we use certainty.

**Flow chart:** 
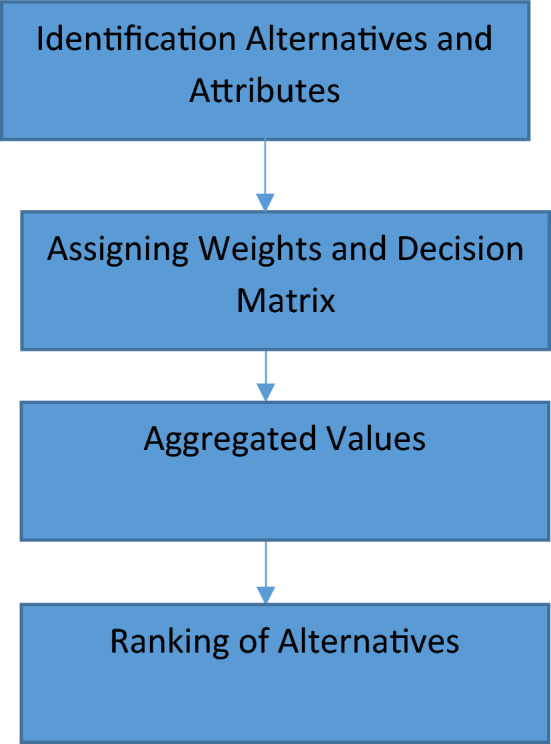


### Application in industrial zone site selection

Using above defined algorithm to select the best alternative (site for industrial zone) among the given alternatives (sites) ${F}_{1},\dots ,{F}_{5}$ on the basis of attributes (i) ${K}_{1}$ is damage to environment; (ii) ${K}_{2}$ is effect on public safety; (iii) ${K}_{3}$ is effect on wildlife safety. Following is the decision matrix decision matrix $D=({d}_{ij}{)}_{5\times 3}$, where entry ${d}_{ij}$ represents the preference of alternative ${F}_{i}(i=1,\dots ,5)$ corresponding to attribute ${K}_{j}(j=\mathrm{1,2},3)$,

$$D=\left[\begin{array}{ccc}0.2& 0.2& 0.4\\ 0.8& 0.8& 0.7\\ 0.3& 0.3& 0.2\\ 0.8& 0.5& 0.6\\ 0.6& 0.7& 0.6\end{array}\right]$$

The weights of the attributes are given as:

$$ w_{1} = \left\langle {\begin{array}{*{20}c} {\left\{ {\left[ {0.3,\,0.7} \right],\left[ {0.2,\,0.4} \right]} \right\},} \\ {\left\{ {\left[ {0.2,\,0.5} \right],\left[ {0.1,\,0.6} \right]} \right\},} \\ {\left\{ {\left[ {0.2,\,0.4} \right],\left[ {0,\,0.1} \right]} \right\},} \\ {\left\{ {0.5,\,0.6} \right\},\left\{ {0.2,\,0.4} \right\},\left\{ {0.2,\,0.3} \right\}} \\ \end{array} } \right\rangle ,w_{2} = \left\langle {\begin{array}{*{20}c} {\left\{ {\left[ {0.5,\,0.7} \right],\left[ {0.2,\,0.5} \right]} \right\},} \\ {\left\{ {\left[ {0.2,\,0.3} \right],\left[ {0.1,\,0.6} \right]} \right\},} \\ {\left\{ {\left[ {0.1,\,0.4} \right],\left[ {0,\,0.3} \right]} \right\},} \\ {\left\{ {0.4,\,0.5} \right\},\left\{ {0.3,\,0.4} \right\},\left\{ {0.2,\,0.4} \right\}} \\ \end{array} } \right\rangle ,w_{3} = \left\langle {\begin{array}{*{20}c} {\left\{ {\left[ {0.4,\,0.5} \right],\left[ {0.6,\,0.7} \right]} \right\},} \\ {\left\{ {\left[ {0.1,\,0.3} \right],\left[ {0.2,\,0.5} \right]} \right\},} \\ {\left\{ {\left[ {0.1,\,0.2} \right],\left[ {0.3,\,0.4} \right]} \right\},} \\ {\left\{ {0.3,\,0.5} \right\},\left\{ {0.4,\,0.6} \right\},\left\{ {0.3,\,0.4} \right\}} \\ \end{array} } \right\rangle $$

The explanation of weights is elaborated as;

In case of ${w}_{1}$, {[0.3,0.7],[0.2,0.4]} is interval hesitant degree of preference for attribute ${K}_{1}$, {[0.2,0.5],[0.1,0.6]} is interval hesitant degree of indeterminacy (preference/ non-preference) for attribute ${K}_{1}$, {[0.2,0.4],[0,0.1]} is interval hesitant degree of non-preference for attribute ${K}_{1}$, {0.1,0.6}} is hesitant degree of preference for attribute ${K}_{1}$, {0.2,0.4} is hesitant degree of indeterminacy (preference/ non-preference) for attribute ${K}_{1}$, {0.4,0.6} is hesitant degree of non-preference for attribute ${K}_{1}$.

#### Aggregated values of alternatives

$$ d_{1} = \left\langle {\begin{array}{*{20}c} {\left\{ {\left[ {0.083651,\,0.240795} \right],\left[ {0.05278,\,0.129345} \right]} \right\},} \\ {\left\{ {\left[ {0.033381,\,0.076327} \right],\left[ {0.026516,\,0.0.174524} \right]} \right\},} \\ {\left\{ {\left[ {0.436991,\,0.8} \right],\left[ {0.382966,\,0.724054} \right]} \right\},} \\ {\left\{ {0.089655,\,0.148579} \right\},\left\{ {0.051616,\,0.100475} \right\},\left\{ {0.675869,\,0.829732} \right\}} \\ \end{array} } \right\rangle $$

$$ d_{2} = \left\langle {\begin{array}{*{20}c} {\left\{ {\left[ {0.617685,\,0.731818} \right],\left[ {0.606713,\,0.702956} \right]} \right\},} \\ {\left\{ {\left[ {0.507612,\,0.596042} \right],\left[ {0.503084,\,0.699876} \right]} \right\},} \\ {\left\{ {\left[ {0.097522,\,0.183507} \right],\left[ {0.101477,\,0.197601} \right]} \right\},} \\ {\left\{ {0.609491,\,0.684432} \right\},\left\{ {0.577689,\,0.663357} \right\},\left\{ {0.158613,\,0.23167} \right\}} \\ \end{array} } \right\rangle $$

$$ d_{3} = \left\langle {\begin{array}{*{20}c} {\left\{ {\left[ {0.089777,\,0.217164} \right],\left[ {0.076525,\,0.164113} \right]} \right\},} \\ {\left\{ {\left[ {0.034223,\,0.07643} \right],\left[ {0.031597,\,0.170692} \right]} \right\},} \\ {\left\{ {\left[ {0.406748,\,0.764784} \right],\left[ {0.382966,\,0.688361} \right]} \right\},} \\ {\left\{ {0.086209,\,0.15133} \right\},\left\{ {0.06256,\,0.123868} \right\},\left\{ {0.648489,\,0.799751} \right\}} \\ \end{array} } \right\rangle $$

$$ d_{4} = \left\langle {\begin{array}{*{20}c} {\left\{ {\left[ {0.445183,\,0.588428} \right],\left[ {0.391659,\,0.530621} \right]} \right\},} \\ {\left\{ {\left[ {0.303378,\,0.385061} \right],\left[ {0.291323,\,0.536908} \right]} \right\},} \\ {\left\{ {\left[ {0.152128,\,0.374173} \right],\left[ {0.142083,\,0.352469} \right]} \right\},} \\ {\left\{ {0.412698,\,0.500953} \right\},\left\{ {\begin{array}{*{20}r} \hfill {0.379002} \\ \end{array} ,\,0.470432} \right\},\left\{ {0.285738,\,0.422203} \right\}} \\ \end{array} } \right\rangle $$

$$ d_{5} = \left\langle {\begin{array}{*{20}c} {\left\{ {\left[ {0.430671,\,0.597105} \right],\left[ {0.407249,\,0.528306} \right]} \right\},} \\ {\left\{ {\left[ {0.315454,\,0.422037} \right],\left[ {0.304401,\,0.547489} \right]} \right\},} \\ {\left\{ {\left[ {0.172143,\,0.361839} \right],\left[ {0.142083,\,0.304007} \right]} \right\},} \\ {\left\{ {0.437361,\,0.528306} \right\},\left\{ {0.381049,\,0.484407} \right\},\left\{ {0.278734,\,0.39362} \right\}} \\ \end{array} } \right\rangle $$

#### *Scores*${S(d}_{i})$

$$S(d_{1} ) = 0.176782,S(d_{2} ) = 0.694794,S(d_{3} ) = 0.19038,S(d_{4} ) = 0.523417,S(d_{5} ) = 0.537091$$.

#### Ranking of alternatives

As $S\left({d}_{2}\right)>S\left({d}_{5}\right)>S\left({d}_{4}\right)>S({d}_{3})>S({d}_{1})$, so that the most desirable alternative is ${F}_{2}$.

**Figure 2:**
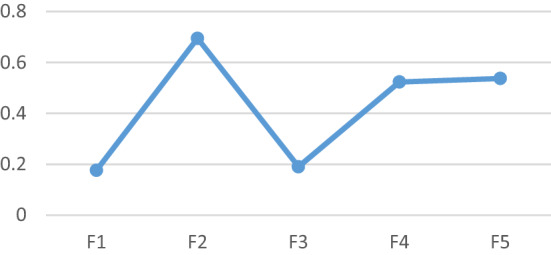
Ranking based on scores.

Figure 2 shows the score function of aggregated values.

Figure 2 elaborate graphically the ordering of aggregated values based on score functions. The alternative ${F}_{2}$ has the highest score and hence is the most desirable or best alternative.

Many ME-MADM methods use the same weights for each attribute corresponding to each decision maker. The following method use different weights for each expert.

**Algorithm 6.3: (ME-MADM problems)**

**Step 1: Identification of alternatives and attributes**

Let $\left\{{F}_{1},{F}_{2},\dots ,{F}_{r}\right\}$ be the set of *r* alternatives, $\left\{{K}_{1},{K}_{2},\dots ,{K}_{s}\right\}$ be *s* attributes. The NCHFS ${\alpha }_{j}$ is used as weight for the attribute ${K}_{j}$. Let $\left\{{E}_{1},{E}_{2},\dots ,{E}_{m}\right\}$ be the decision experts. The decision matrix is ${D}^{(k)}=({{d}_{ij}}^{(k)})$ consisting fuzzy values, where ${d}_{ij}$ represent the preference given by the kth expert ${E}_{k}$ to alternative ${F}_{i}$ corresponding to attribute ${K}_{j}$.

**Step 2. Allocation of weights to attributes**

The NCHF value ${{\alpha }_{j}}^{(k)}$ is used as weight assigned to attribute ${K}_{j}$ by expert ${E}_{k}$.

**Step 3: Computation of weighted aggregated values**

Using NCHFWEA operators, compute the aggregated values ${{d}_{j}}^{(k)}{^{\prime}}s$ (*j* = *1,…,r; k* = *1,..m*) of alternatives ${F}_{j}{^{\prime}}s$ on the bases of weights assigned by experts.

**Step 4: Transformations of** ${{{\varvec{d}}}_{{\varvec{j}}}}^{({\varvec{k}})}{^{\prime}}{\varvec{s}}$ **to** ${{\varvec{d}}}_{{\varvec{j}}}{^{\prime}}{\varvec{s}}$

The transformation is based on the formula ${d}_{j}={{u}_{1}}^{{{d}_{j}}^{(1)}}\otimes \dots \otimes {{u}_{m}}^{{{d}_{j}}^{(m)}}$, where ${u}_{k}$ (*k* = *1,..m*) is the weight assigned to expert ${E}_{k}$.

**Step 5. Ranking of Alternatives**

We calculate the scores ${S(d}_{j});j=1,\dots ,r$ of the aggregated values. Using scores ${S(d}_{i});i=1,\dots ,n$,we rank the alternatives ${F}_{i};i=1,\dots ,n$. If scores of two alternatives are equal, then we use accuracy function for ranking and if they have same accuracy, we use certainty.

**Flow Chart:** 
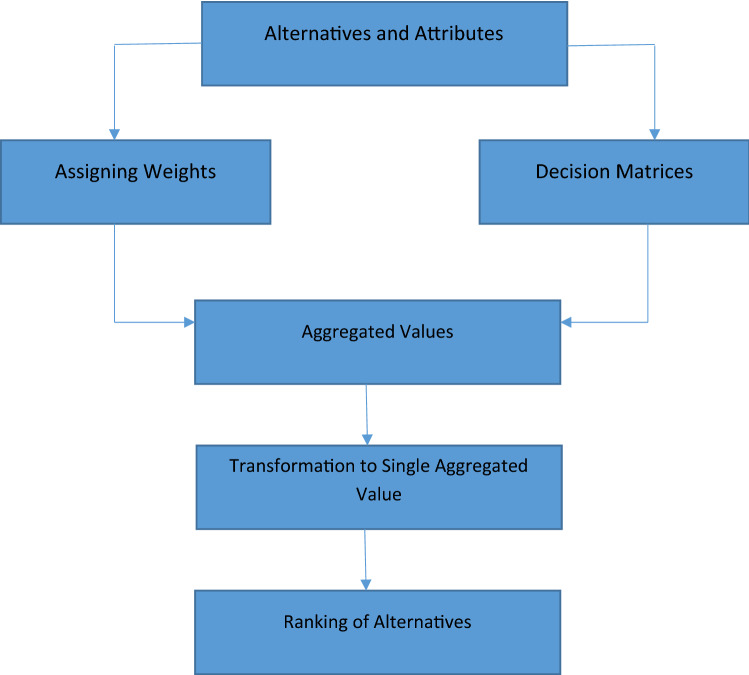


### Solid waste disposal site selection

Using above defined algorithm to select the best alternative (solid waste disposal site) among the given alternatives (sites) ${F}_{1},\dots ,{F}_{4}$ on the basis of attributes (i) ${K}_{1}$ is water pollution; (ii) ${K}_{2}$ slope; (iii) ${K}_{3}$ distance from residential area. Following are the decision matrices ${D}^{(k)}=({d}_{ij}{)}_{4\times 3}$, where entry ${d}_{ij}$ represents the preference given by the expert ${E}_{k}$ *(k* = *1,2)* to ${F}_{i}(i=1,\dots ,4)$ corresponding to ${K}_{j}(j=\mathrm{1,2},3)$.

$${D}_{1}=\left[\begin{array}{ccc}0.5& 0.4& 0.3\\ 0.6& 0.6& 0.3\\ 0.7& 0.8& 0.5\\ 0.7& 0.5& 0.4\end{array}\right],{D}_{2}=\left[\begin{array}{ccc}0.6& 0.3& 0.5\\ 0.5& 0.4& 0.4\\ 0.8& 0.6& 0.7\\ 0.6& 0.6& 0.5\end{array}\right]$$

The weights for attributes given by experts are:

$$ w_{1}^{(1)} = \left\langle {\begin{array}{*{20}c} {\left\{ {\left[ {0.4,\,0.7} \right],\left[ {0.3,0.5} \right]} \right\},} \\ {\left\{ {\left[ {0.2,\,0.5} \right],\left[ {0.1,\,0.6} \right]} \right\},} \\ {\left\{ {\left[ {0.2,\,0.4} \right],\left[ {0,\,0.1} \right]} \right\},} \\ {\left\{ {0.6,\,0.7} \right\},\left\{ {0.2,\,0.3} \right\},\left\{ {0.1,\,0.3} \right\}} \\ \end{array} } \right\rangle ,w_{2}^{(1)} = \left\langle {\begin{array}{*{20}c} {\left\{ {\left[ {0.3,\,0.5} \right],\left[ {0.6,\,0.7} \right]} \right\},} \\ {\left\{ {\left[ {0.2,\,0.3} \right],\left[ {0.1,\,0.6} \right]} \right\},} \\ {\left\{ {\left[ {0.1,\,0.4} \right],\left[ {0,\,0.3} \right]} \right\},} \\ {\left\{ {0.5,\,0.7} \right\},\left\{ {0.1,\,0.3} \right\},\left\{ {0.3,\,0.4} \right\}} \\ \end{array} } \right\rangle ,w_{3}^{(1)} = \left\langle {\begin{array}{*{20}c} {\left\{ {\left[ {0.4,\,0.5} \right],\left[ {0.6,\,0.7} \right]} \right\},} \\ {\left\{ {\left[ {0.1,\,0.3} \right],\left[ {0.2,\,0.5} \right]} \right\},} \\ {\left\{ {\left[ {0.1,\,0.3} \right],\left[ {0.4,\,0.5} \right]} \right\},} \\ {\left\{ {0.4,\,0.6} \right\},\left\{ {0.1,\,0.2} \right\},\left\{ {0.2,\,0.3} \right\}} \\ \end{array} } \right\rangle $$

$$ w_{1}^{(2)} = \left\langle {\begin{array}{*{20}c} {\left\{ {\left[ {0.4,\,0.6} \right],\left[ {0.3,\,0.4} \right]} \right\},} \\ {\left\{ {\left[ {0.3,\,0.4} \right],\left[ {0.1,\,0.2} \right]} \right\},} \\ {\left\{ {\left[ {0.2,\,0.5} \right],\left[ {0,\,0.1} \right]} \right\},} \\ {\left\{ {0.4,\,0.6} \right\},\left\{ {0.2,\,0.4} \right\},\left\{ {0.3,\,0.5} \right\}} \\ \end{array} } \right\rangle ,w_{2}^{(2)} = \left\langle {\begin{array}{*{20}c} {\left\{ {\left[ {0.2,\,0.4} \right],\left[ {0.5,\,0.6} \right]} \right\},} \\ {\left\{ {\left[ {0.2,\,0.4} \right],\left[ {0.3,\,0.5} \right]} \right\},} \\ {\left\{ {\left[ {0.2,\,0.4} \right],\left[ {0,\,0.3} \right]} \right\},} \\ {\left\{ {0.7,\,0.8} \right\},\left\{ {0.2,\,0.3} \right\},\left\{ {0.4,\,0.5} \right\}} \\ \end{array} } \right\rangle ,w_{3}^{(2)} = \left\langle {\begin{array}{*{20}c} {\left\{ {\left[ {0.3,\,0.5} \right],\left[ {0.6,\,0.7} \right]} \right\},} \\ {\left\{ {\left[ {0.1,\,0.2} \right],\left[ {0.3,\,0.6} \right]} \right\},} \\ {\left\{ {\left[ {0.1,\,0.4} \right],\left[ {0.3,\,0.5} \right]} \right\},} \\ {\left\{ {0.5,\,0.6} \right\},\left\{ {0.1,\,0.3} \right\},\left\{ {0.4,\,0.5} \right\}} \\ \end{array} } \right\rangle $$

The explanation of weights is elaborated as;

In case of ${{w}_{1}}^{(2)}$, {[0.4,0.6],[0.3,0.4]} is interval hesitant degree of preference to attribute ${K}_{1}$, {[0.3,0.4],[0.1,0.2]} is interval hesitant degree of indeterminacy (preference/ non-preference) for attribute ${K}_{1}$, {[0.2,0.5],[0,0.1]} is interval hesitant degree of non-preference for attribute ${K}_{1}$, {0.4,0.6}} is hesitant degree of preference for attribute ${K}_{1}$, {0.2,0.4} is hesitant degree of indeterminacy (preference/ non-preference) for attribute ${K}_{1}$, {0.3,0.5} is hesitant degree of non-preference for attribute ${K}_{1}$, given by second expert.

#### Aggregated values of alternatives

$$ d_{1}^{(1)} = \left\langle {\begin{array}{*{20}c} {\left\{ {\left[ {0.168693,\,0.281372} \right],\left[ {0.263604,\,0.374317} \right]} \right\},} \\ {\left\{ {\left[ {0.093376,\,0.160292} \right],\left[ {0.089665,\,0.287722} \right]} \right\},} \\ {\left\{ {\left[ {0.295774,\,0.633943} \right],\left[ {0.382199,\,0.611782} \right]} \right\},} \\ {\left\{ {0.232751,\,0.381204} \right\},\left\{ {0.093376,\,0.123714} \right\},\left\{ {0.442892,\,0.60767} \right\}} \\ \end{array} } \right\rangle $$

$$ d_{2}^{(1)} = \left\langle {\begin{array}{*{20}c} {\left\{ {\left[ {0.249959,\,0.363983} \right],\left[ {0.352221,\,0.463081} \right]} \right\},} \\ {\left\{ {\left[ {0.149435,\,0.23322} \right],\left[ {0.152184,\,0.363983} \right]} \right\},} \\ {\left\{ {\left[ {0.239398,\,0.536919} \right],\left[ {0.382199,\,0.5535} \right]} \right\},} \\ {\left\{ {0.306626,\,0.454715} \right\},\left\{ {0.149435,\,0.186685} \right\},\left\{ {0.359256,\,0.512649} \right\}} \\ \end{array} } \right\rangle $$

$$ d_{3}^{(1)} = \left\langle {\begin{array}{*{20}c} {\left\{ {\left[ {0.455621,\,0.568276} \right],\left[ {0.539999,\,0.635575} \right]} \right\},} \\ {\left\{ {\left[ {0.336995,\,0.440546} \right],\left[ {0.340836,\,0.560738} \right]} \right\},} \\ {\left\{ {\left[ {0.150378,\,0.355881} \right],\left[ {0.242142,\,0.361851} \right]} \right\},} \\ {\left\{ {0.511642,\,0.636861} \right\},\left\{ {0.336995,\,0.382743} \right\},\left\{ {0.214347,\,0.332492} \right\}} \\ \end{array} } \right\rangle $$

$$ d_{4}^{(1)} = \left\langle {\begin{array}{*{20}c} {\left\{ {\left[ {0.286796,\,0.401832} \right],\left[ {0.409242,\,0.516248} \right]} \right\},} \\ {\left\{ {\left[ {0.189282,\,0.271188} \right],\left[ {0.186771,\,0.415582} \right]} \right\},} \\ {\left\{ {\left[ {0.207275,\,\begin{array}{*{20}r} \hfill {0.500834} \\ \end{array} } \right],\left[ {0.306855,\,0.504286} \right]} \right\},} \\ {\left\{ {0.353802,\,0.505876} \right\},\left\{ {\begin{array}{*{20}r} \hfill {0.189282} \\ \end{array} ,\,0.230407} \right\},\left\{ {0.347451,\,0.482708} \right\}} \\ \end{array} } \right\rangle $$

$$ d_{1}^{(2)} = \left\langle {\begin{array}{*{20}c} {\left\{ {\left[ {0.172929,\,0.27991} \right],\left[ {0.290305,\,0.369343} \right]} \right\},} \\ {\left\{ {\left[ {0.143046,\,0.205277} \right],\left[ {0.16734,\,0.275848} \right]} \right\},} \\ {\left\{ {\left[ {0.337857,\,0.661617} \right],\left[ {0.187748,\,0.531795} \right]} \right\},} \\ {\left\{ {0.36267,\,0.485593} \right\},\left\{ {0.135922,\,0.195054} \right\},\left\{ {0.598318,\,0.7} \right\}} \\ \end{array} } \right\rangle $$

$$ d_{2}^{(2)} = \left\langle {\begin{array}{*{20}c} {\left\{ {\left[ {0.166906,\,0.276601} \right],\left[ {0.269857,0.347395} \right]} \right\},} \\ {\left\{ {\left[ {0.129653,\,0.182922} \right],\left[ {0.148579,\,0.251785} \right]} \right\},} \\ {\left\{ {\left[ {0.338679,\,0.667768} \right],\left[ {0.240342,\,0.551725} \right]} \right\},} \\ {\left\{ {0.316979,\,\begin{array}{*{20}r} \hfill {0.437345} \\ \end{array} } \right\},\left\{ {0.12097,\,0.182922} \right\},\left\{ {0.609754,\,0.717157} \right\}} \\ \end{array} } \right\rangle $$

$$ d_{3}^{(2)} = \left\langle {\begin{array}{*{20}c} {\left\{ {\left[ {0.452839,\,0.563217} \right],\left[ {0.574484,\,0.640684} \right]} \right\},} \\ {\left\{ {\left[ {0.412356,\,0.483975} \right],\left[ {0.445713,\,0.561807} \right]} \right\},} \\ {\left\{ {\left[ {0.166783,\,0.385742} \right],\left[ {0.101477,\,0.298055} \right]} \right\},} \\ {\left\{ {0.627839,\,0.715988} \right\},\left\{ {0.403256,\,0.476572} \right\},\left\{ {0.338962,\,0.420345} \right\}} \\ \end{array} } \right\rangle $$

$$ d_{4}^{(2)} = \left\langle {\begin{array}{*{20}c} {\left\{ {\left[ {0.301086,\,0.424264} \right],\left[ {0.410553,\,0.487351} \right]} \right\},} \\ {\left\{ {\left[ {0.249058,\,0.311141} \right],\left[ {0.186771,\,0.415582} \right]} \right\},} \\ {\left\{ {\left[ {0.239398,\,\begin{array}{*{20}r} \hfill {0.521454} \\ \end{array} } \right],\left[ {0.187748,\,0.423571} \right]} \right\},} \\ {\left\{ {0.4465,\,0.5578} \right\},\left\{ {\begin{array}{*{20}r} \hfill {0.236655} \\ \end{array} ,\,0.316866} \right\},\left\{ {0.469978,\,0.575736} \right\}} \\ \end{array} } \right\rangle $$

#### Transformed aggregated values

Using weights ${u}_{1}={u}_{2}=0.5$ for experts, we have

$$ d_{1} = \left\langle {\begin{array}{*{20}c} {\left\{ {\left[ {0.316795,\,0.368895} \right],\left[ {0.367014,\,0.418605} \right]} \right\},} \\ {\left\{ {\left[ {0.294517,\,0.322097} \right],\left[ {0.298749,\,0.36948} \right]} \right\},} \\ {\left\{ {\left[ {0.534735,\,0.62635} \right],\left[ {0.543718,\,0.607142} \right]} \right\},} \\ {\left\{ {0.377728,\,0.455903} \right\},\left\{ {0.293066,\,0.311816} \right\},\left\{ {0.598383,\,0.633952} \right\}} \\ \end{array} } \right\rangle $$

$$ d_{2} = \left\langle {\begin{array}{*{20}c} {\left\{ {\left[ {0.333756,\,0.38974} \right],\left[ {0.384772,\,0.438448} \right]} \right\},} \\ {\left\{ {\left[ {0.303357,\,0.333589} \right],\left[ {0.307949,\,0.383094} \right]} \right\},} \\ {\left\{ {\left[ {0.484897,\,0.655379} \right],\left[ {0.543036,\,0.579802} \right]} \right\},} \\ {\left\{ {0.38518,\,0.463956} \right\},\left\{ {0.301537,\,0.323} \right\},\left\{ {0.572655,\,0.611645} \right\}} \\ \end{array} } \right\rangle $$

$$ d_{3} = \left\langle {\begin{array}{*{20}c} {\left\{ {\left[ {0.46926,\,0.547713} \right],\left[ {0.541293,\,0.605525} \right]} \right\},} \\ {\left\{ {\left[ {0.420259,\,0.474513} \right],\left[ {0.431236,\,0.544327} \right]} \right\},} \\ {\left\{ {\left[ {0.463809,\,0.646651} \right],\left[ {0.50664,\,0.558764} \right]} \right\},} \\ {\left\{ {0.550754,\,0.63854} \right\},\left\{ {0.417617,\,0.453544} \right\},\left\{ {0.560708,\,0.59502} \right\}} \\ \end{array} } \right\rangle $$

$${d_4} = \left\langle {\begin{array}{*{20}{c}}
{\left\{ {\left[ {0.445183,\,0.588428} \right],\left[ {0.391659,\,0.530621} \right]} \right\},}\\
{\left\{ {\left[ {0.303378,\,0.385061} \right],\left[ {0.291323,\,0.536908} \right]} \right\},}\\
{\left\{ {\left[ {0.152128,\,0.374173} \right],\left[ {0.142083,\,0.352469} \right]} \right\},}\\
{\left\{ {0.412698,\,0.500953} \right\},\left\{ {\begin{array}{*{20}{r}}
{0.379002}
\end{array},\,0.470432} \right\},\left\{ {0.285738,\,0.422203} \right\}}
\end{array}} \right\rangle 
$$

#### Scores ${S(d}_{i})$

$$S\left({d}_{1}\right)=0.369,S\left({d}_{2}\right)=0.383,S\left({d}_{3}\right)=0.503,S\left({d}_{4}\right)=0.420$$.

#### Ranking of alternatives

As $S\left({d}_{3}\right)>S\left({d}_{4}\right)>S({d}_{2})>S({d}_{1})$, so that the most desirable alternative is ${F}_{3}$.

**Figure 3:**
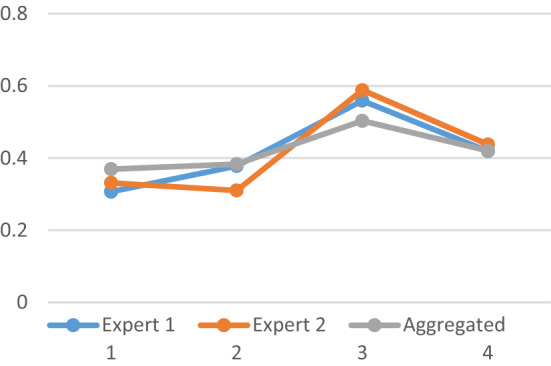
Ranking based on scores.

Figure 3 shows the score function of expert aggregated values and transformed aggregated.

The Fig. 3 is a graphical reflection of scores of aggregated values corresponding to each expert and transformed aggregated values. The figure reflects that the alternative ${F}_{3}$ has the highest score of transformed aggregated value and hence the most desirable or best alternative while ${F}_{1}$ is the worse alternative.

### Comparative analysis

The industrial zone site selection problem is solved by some existing techniques and findings are presented in the following table.

Table 1 indicate that the proposed method agrees with existing methods in all alternatives. This also validate the validity of proposed method as well.

**Table 1:** Comparison of the results.

Method	Scores S, cosine similarities C	Ranking
Lu and Ye ^44^	$S(d_{1} ) = 0.454057$, $S(d_{2} ) = 0.624296$, $S(d_{3} ) = 0.458387$, $S(d_{4} ) = 0.582653$, $S(d_{5} ) = 0.592526$	${F}_{2}>{F}_{5}>{F}_{4}>{F}_{3}>{F}_{1}$
Tan et al., ^45^	$S(d_{1} ) = - 0.28165$, $S(d_{2} ) = 0.61758$, $S(d_{3} ) = - 0.25181$, $S(d_{4} ) = 0.379552$, $S(d_{5} ) = 0.400404$	${F}_{2}>{F}_{5}>{F}_{4}>{F}_{3}>{F}_{1}$
Ye ^43^	$C(d_{1} ) = 0.495585$, $C(d_{2} ) = 0.948553$, $C(d_{3} ) = 0.514484$, $C(d_{4} ) = 0.867698$, $C(d_{5} ) = 0.87221$	${F}_{2}>{F}_{5}>{F}_{4}>{F}_{3}>{F}_{1}$
Current study	$S(d_{1} ) = 0.176782$, $S(d_{2} ) = 0.694794$, $S(d_{3} ) = 0.19038$, $S(d_{4} ) = 0.523417$, $S(d_{5} ) = 0.537091$	${F}_{2}>{F}_{5}>{F}_{4}>{F}_{3}>{F}_{1}$

## Conclusion

In this study, first we proposed exponential operational laws in NCHFS and investigates the fundamental properties of these exponential laws. Using these exponential laws, the exponential aggregation operators are proposed in the environment of NCHFS, which is a useful addition in the family of aggregation operators. Then we established a method to solve complex ME-MADM problem where each expert has its own decision matrix along with his own weighting vector for attributes. Finally, the proposed method is applied to the industrial zone site selection and solid waste disposal site selections are problems.

## Data Availability

The datasets generated and/or analyzed during the current study does not use any specific data.
